# Unified long-read panel for Parkinson’s and repeat expansion disorders

**DOI:** 10.1038/s41531-026-01585-4

**Published:** 2026-09-24

**Authors:** André Fienemann, Julia C. Prietzsche, Joshua Laβ, Christoph Much, Susen Schaake, Theresa Lüth, Carolin Gabbert, Alexander Zimprich, Elisabeth Stögmann, Theresa König, Christos Ganos, Tuğçe Gül-Demirkale, A. Nazlı Başak, Roland Dominic G. Jamora, Raymond L. Rosales, Gerard Saranza, Cid Czarina E. Diesta, Meret Möller, Max Borsche, Teresa Kleinz, Alexander Balck, Norbert Brüggemann, Philip Seibler, Christine Klein, Joanne Trinh

**Affiliations:** 1 Institute of Neurogenetics, University of Lübeck, Lübeck, Germany; 2 Department of Neurology, Medical University of Vienna, Vienna, Austria; 3 Edmond J. Safra Program in Parkinson’s Disease and the Morton and Gloria Shulman Movement Disorders Clinic, Toronto Western Hospital, Toronto, ON Canada; 4 Division of Neurology, University of Toronto, Toronto, ON Canada; 5 Krembil Brain Institute, Toronto, ON Canada; 6 Suna and İnan Kıraç Foundation, Neurodegeneration Research Laboratory (NDAL), KUTTAM, School of Medicine, Koç University, İstanbul, Turkey; 7 Department of Neurosciences, College of Medicine-Philippine General Hospital, University of the Philippines Manila, Manila, Philippines; 8 Department of Neurology and Psychiatry, University of Santo Tomas and the CNS-Metropolitan Medical Center, Manila, Philippines; 9 Section of Neurology, Department of Internal Medicine, Chong Hua Hospital, Cebu, Philippines; 10 Department of Neurosciences, Movement Disorders Clinic, Makati Medical Center, Makati City, Philippines; 11 Section for Movement Disorders, Department of Neurology, University of Lübeck and University Hospital Schleswig-Holstein, Lübeck, Germany

**Keywords:** Diseases, Genetics, Neurology, Neuroscience

## Abstract

In this study, we designed a gene panel based on Nanopore long-read sequencing using adaptive sampling, targeting *n* = 564 genes associated with Parkinson’s disease (PD) and repeat expansion disorders. We investigated its diagnostic utility in *n* = 18 patients with (1) pathogenic variants in *LRRK2*, *PRKN*, *SNCA*, and *RAB32* (*n* = 7); (2) idiopathic PD or FTD negative for known genetic causes (*n* = 3); (3) repeat expansions in *ATXN1*, *ATXN2*, *ATXN3*, *C9orf72*, *DAB1*, *FGF14*, *HTT*, and *TAF1*-SVA (*n* = 8). Three individuals were multiplexed per flow cell, achieving a mean coverage of 24X (SD = ± 9X) per sample. Ultimately, 17/20 (85%; Clopper–Pearson 95% CI: 62–97%) expected pathogenic variants were identified; i.e., an *SNCA* triplication and two repeat expansions in *C9orf72* and *TAF1*-SVA were missed. Additional variants in *STXBP2*, *AP4S1*, *RARS2*, *ALS2*, and *CNBP* were identified in five patients. Adaptive sampling is a versatile genetic diagnostic tool in neurodegenerative disorders, while enabling cost-effective multiplexing. Further validation and improvements in bioinformatic analysis are needed.

## Introduction

Parkinson’s disease (PD) is the second-largest and fastest-growing neurodegenerative disorder, with global patient numbers expected to increase by 112% from 2021 to 2050, primarily due to an aging population^1–3^. The diagnostic criteria for PD are currently still based on motor features caused by neurodegeneration^4–6^. The diagnostic process can be challenging due to the heterogeneity of signs and symptoms and is supported by pathologic presynaptic dopaminergic imaging. At present, the α-synuclein seed amplification assay (α-syn SAA)^7^ is not yet approved for the clinical diagnosis of PD, but it is used in research and is advancing efforts in biomarker development. Genetic testing is increasingly used to aid diagnosis and provide insights into treatment responsiveness and familial risk^8,9^. Genetic variants associated with PD can be identified in up to 15% of patients; however, variable penetrance and variants of unknown significance (VUS) complicate testing^5,10,11^.

The clinical features and family history often inform the choice of genetic test^12,13^, with sequential assays being performed if the genetic cause cannot be determined, e.g., exome sequencing, followed by multiplex ligation-dependent probe amplification (MLPA) to detect structural variants (SVs)^14^. This can be costly and time‑consuming. Exploring new methods for genetic screening is warranted, as costs and access to genetic testing vary by region and clinical setting^5,15^.

**Fig. 1:**
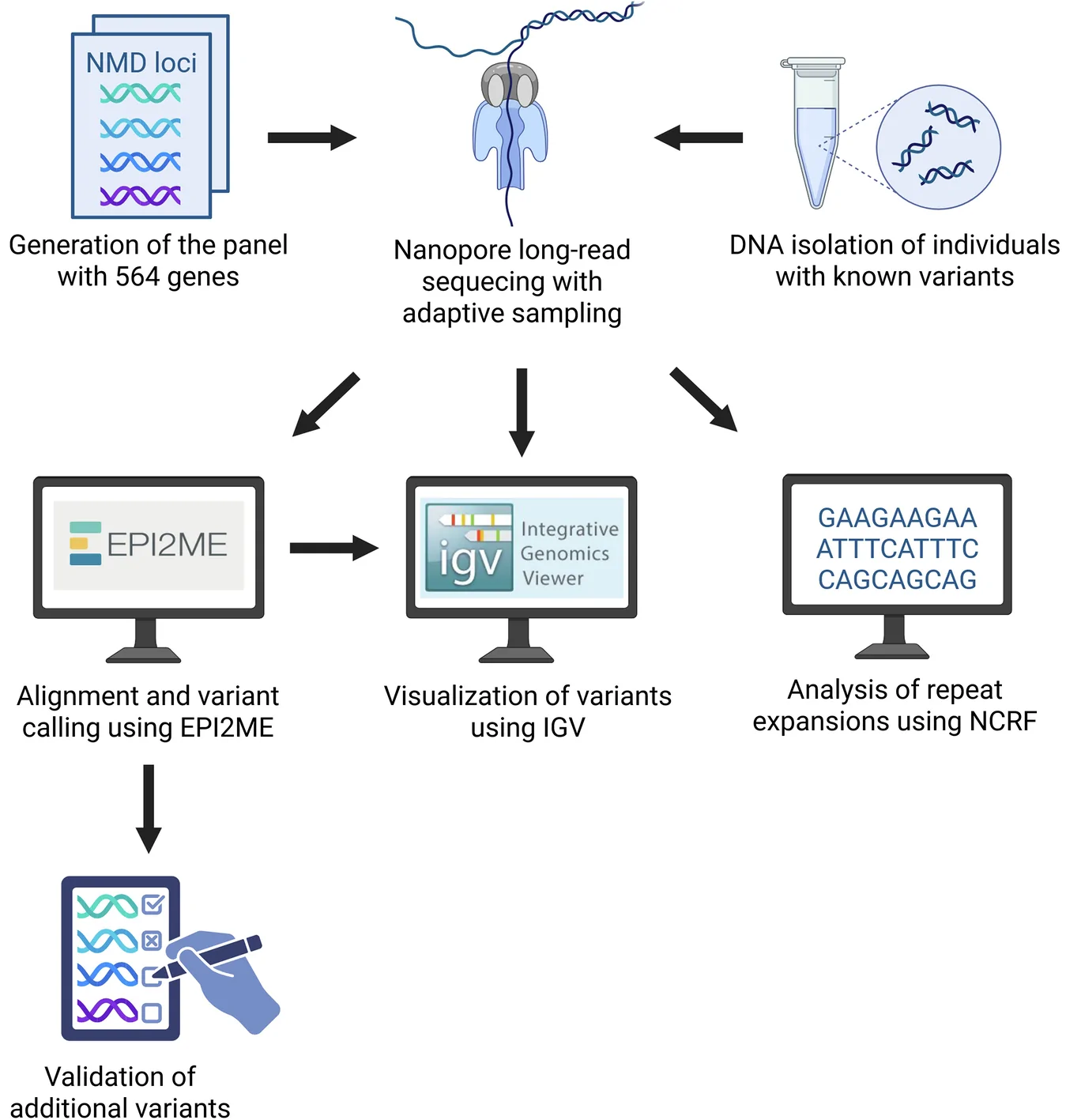
Overview of the workflow to evaluate the panel’s diagnostic utility on samples with known variants. A gene panel for neurodegenerative movement disorders (NMDs) was created, consisting of 564 loci linked to Parkinson’s disease and repeat expansion disorders. DNA was isolated from *n* = 18 individuals with known genetic variants and sequenced using ONT long-read sequencing to test this panel’s diagnostic utility. Adaptive sampling was applied during the sequencing to enrich the gene loci of the panel. The human variation workflow from EPI2ME Labs was used to align reads and call variants. The visualization of the aligned reads was performed by the Integrative Genomics Viewer (IGV). The Noise Cancelling Repeat Finder (NCRF) was used to analyze the repeat expansion loci not included in the EPI2ME human variation workflow. Additional pathogenic variants detected by the EPI2ME human variation workflow were validated using PCR-based Sanger sequencing for SNVs and indels or agarose gel electrophoresis for SVs and repeat expansions. ONT: Oxford Nanopore Technologies, SNV: Single-nucleotide variant. This figure was created in Created in BioRender. Fienemann, A. (2026) https://BioRender.com/hwptm5k.

Novel sequencing techniques have significantly advanced genetic research, from Sanger sequencing to Next Generation Sequencing (NGS) using short reads, and now to long-read sequencing (LRS)^16,17^. LRS has enabled the discovery of multiple repeat expansion disorder loci^18,19^ and can sequence challenging regions, including large SVs and repetitive regions, such as short tandem repeats (STRs)^20–22^. Oxford Nanopore Technologies (ONT) specifically offers targeted LRS called adaptive sampling, which is based on mapping actively sequenced reads to predefined regions of interest. Only reads aligning to those regions are completely sequenced. In contrast, off-target reads are ejected from the Nanopore, resulting in targeted enrichment and deeper coverage of predefined targets, e.g., disease-relevant genes. Adaptive sampling has been previously used for gene panels with the potential to improve diagnostic rates for repeat expansion disorders^18,23,24^, and has shown promise in detecting diverse pathogenic variant types^25^. While the use of adaptive sampling for the detection of single-nucleotide variants (SNVs), indels, and repeat expansions has been investigated, especially in ataxias^18,23^, other movement disorders with pathogenic variants, i.e., large structural variants, remain understudied. Here, we designed a novel gene panel comprising 564 loci related to PD, repeat expansion disorders, and other neurodegenerative and movement disorders, and performed adaptive sampling to jointly interrogate phased SNVs, indels, large SVs, and repeat expansions. We investigate the technical feasibility of our approach and its potential diagnostic utility (Fig. 1).

## Results

### Read quality and coverage of the gene panel

**Fig. 2:**
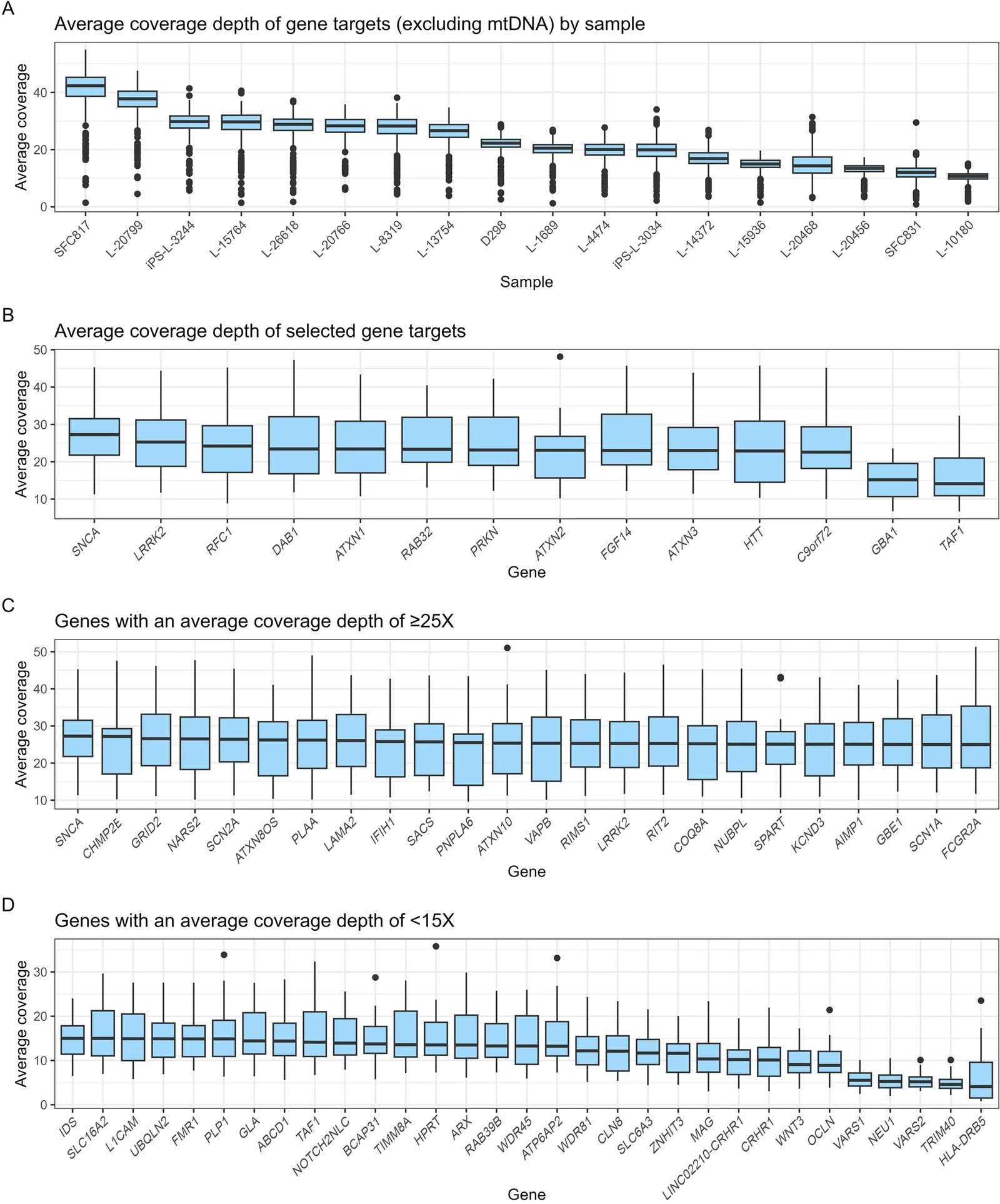
Sequencing coverage across genes for the targeted long-read sequencing panel. Average coverage depth of gene targets obtained for **A** each sample, **B** the samples obtained for selected gene targets on the targeted long-read sequencing panel, **C** the gene targets with an average coverage of ≥25X, and **D** the gene targets with an average coverage of <15X. The mtDNA was excluded from the coverage analyses.

The average number of mapped on-target reads per sample was 420,408 (SD = ± 244,251) (Table 1, Supplementary Table 2). Across all samples, a mean Phred quality score of 19.7 (SD = ± 2.2) was achieved. The mean on-target read N50 of all samples was 21.5 kb (SD = ± 11 kb). Across all samples, a mean on-target coverage of 24X (SD = ± 9X) was achieved. The coverage of all 563 loci of the gene panel (excluding the mtDNA) differed both between samples (Fig. 2A) and across the individual gene loci (Fig. 2B). In particular, the genes affected by known pathogenic variants (Table 2) showed differences in the mean coverage depths (Fig. 2C). *SNCA* and *LRRK2* were among the genes with a mean coverage of ≥25X (Fig. 2B, C), while *GBA1* and *TAF1* showed a mean coverage of approximately 15X (Fig. 2B, D). The remaining selected gene targets exhibited coverage ranging from 23–28X (Fig. 2B). For the mtDNA, a mean coverage of 1256X was obtained.

**Table 1:** Read statistics from the ONT long-read adaptive sampling runs

	Total mapped on-target reads	On-target coverage (X)	Quality (*Q*-score)	Read length (kb)	On-target Read N50 (kb)
Mean	418,989	24.0	19.6	5.37	21.5
Median	382,251	21.7	19.4	4.72	18.6
SD	251,251	9.0	2.2	3.49	11.3
IQR	380,679	12.5	3.4	4.32	20.1

Descriptive statistics were calculated based on all 18 samples of the panel.

*SD* standard deviation, *IQR* interquartile range, *kb* kilobase, *Q*-*score* read quality (Phred), *X* depth of coverage.

**Table 2:** Patient demographics and types of prior genetic testing for the gene panel for Parkinson’s disease and repeat expansion disorders

Sample ID	Sex	AAO	AAE	Known pathogenic variant	Prior genetic testing
L-15936	female	*NA*	44	*GBA1* c.1223C > T *LRRK2* c.6055G > A (p.G2019S)	Sanger sequencing
iPS-L-3244	female	39	45	*PRKN* c.823C > T, *PRKN* ex1del	OGM, LRS
SFC817	male	64	75	*PRKN* c.971del, *PRKN* ex7del	OGM, LRS
L-13754	female	29	71	*PRKN* ex2-3del, *PRKN* c.101_102del	MLPA
iPS-L-3034	male	38	53	*PRKN* ex2del, *PRKN* ex3-5del	OGM, LRS
L-26618	male	53	60	*RAB32* c.213C > G	NGS gene panel, WGS
SFC831	female	35-40	55	*SNCA* triplication	OGM, LRS
L-20456	male	33	46	*ATXN1* repeat expansion	PCR-based LRS
L-20468	male	41	44	*ATXN2* repeat expansion	PCR-based LRS
L-10180	female	46	52	*ATXN3* repeat expansion	PCR-based LRS
L-20677	male	65	67	*HTT* repeat expansion	*NA*
L-1689	female	15	80	*DAB1* repeat expansion	PCR-based LRS
L-15764	male	63	70	*FGF14* repeat expansion	PCR-based LRS
L-20799	male	50	60	*C9orf72* repeat expansion	Repeat-primed PCR
L-14372	male	41	46	*TAF1*-SVA repeat expansion	PCR-based LRS
D298	female	58	59	None (idiopathic FTD)	WES, Repeat-primed PCR
L-4474	female	47	62	None (idiopathic PD)	OGM, LRS
L-8319	male	*NA*	31	None (idiopathic PD)	*NA*

*AAO* Age At Onset, *AAE* Age At Examination, *FTD* Frontotemporal Dementia, *LRS* Long-read Sequencing, *MLPA* Multiplex Ligation-dependent Probe Amplification, *NA* not applicable or not available, *NGS* Next Generation Sequencing, *OGM* Optical Genome Mapping, *PCR* Polymerase Chain Reaction, *PD* Parkinson’s Disease, *WES* Whole Exome Sequencing, *WGS* Whole Genome Sequencing.

### Detection of the known pathogenic variants with EPI2ME

The human variation workflow from the ONT bioinformatics software collection, EPI2ME, was applied as a screening tool to validate the performance of the gene panel using pathogenic variants detected by prior genetic testing in eighteen individuals from established patient cohorts (Table 2). This includes SNVs, indels, SVs, and repeat expansions.

**Fig. 3:**
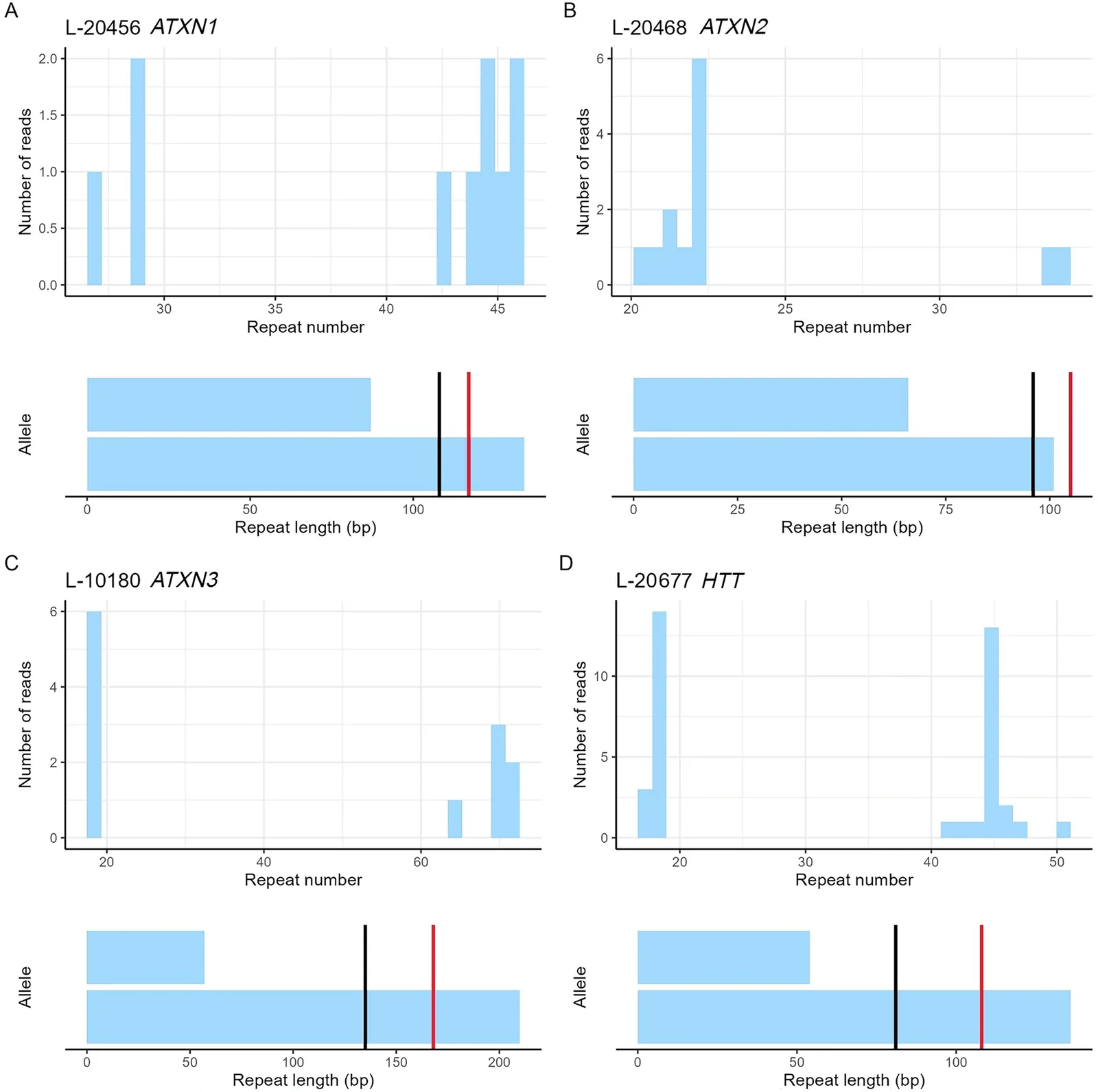
Histograms of the repeat expansions detected using EPI2ME. **A**. *ATXN1* (CAG)_45_ repeat expansion in L-20456. **B**. *ATXN2* (CAG)_34_ repeat expansion in L-20468. **C**. *ATXN3* (CAG)_70_ repeat expansion in L-10180. **D**. *HTT* (CAG)_45_ repeat expansion in L-20677. For each repeat expansion, the top panel shows the histogram with the number of reads for each repeat length based on the number of repetitions of the repeat motif. The bottom panel shows the repeat length of the short and expanded allele in base pairs (bp) with the intermediate (black line) and pathogenic threshold (red line).

Regarding the detection of SNVs and indels, the small variant caller Clair3 in the EPI2ME workflow correctly detected all six pathogenic small variants: the c.1223C > T (p.T408M) *GBA1* variant and the c.6055G > A (p.G2019S) *LRRK2* variant in L-15936, the three *PRKN* variants c.823C > T (p.R275W), c.971del (p.V324fs), and c.101_102del (p.Q34fs) in iPS-L-3244, SFC817, and L-13754, and the c.213C > G (p.S71R) *RAB32* variant in L-26618 (Table 3). The small variant caller BCFtools, applied after the initial analysis, also confirmed all six variants. Regarding the detection of SVs, the SV caller Sniffles2, included in the EPI2ME workflow, correctly detected two large *PRKN* deletions (≥200 kb) in SFC817 and iPS-L-3034 with default parameters (–long-del-length: 50000, –long-del-coverage: 0.66). However, it failed to identify three additional *PRKN* deletions in iPS-L-3244, L-13754, and iPS-L-3034, as well as a 1.7 Mb triplication encompassing the entire *SNCA* gene in SFC831 (Table 3). For SFC817, the read-based phasing software Whatshap confirmed compound heterozygosity of the two *PRKN* variants (c.971del and ex7del). Using adjusted parameters to account for the detection of SVs >100 kb, both CuteSV and SVIM, two SV callers applied after the initial analysis, showed performance equal to Sniffles2, with CuteSV additionally detecting the two missed *PRKN* deletions in L-13754 (320 kb) and iPS-L-3034 (112 kb). Regarding the detection of repeat expansions, the tandem repeat variant caller Straglr, included in the EPI2ME workflow, correctly identified four pathogenic repeat expansions: an *ATXN1* (CAG)_45_ repeat expansion in L-20456 (Fig. 3A), an *ATXN2* (CAG)_34_ repeat expansion in L-20468 (Fig. 3B), an *ATXN3* (CAG)_70_ repeat expansion in L-10180 (Fig. 3C), and an *HTT* (CAG)_45_ repeat expansion in L-20677 (Fig. 3D). The expected repeat number (RN) based on prior genetic testing was 44 for *ATXN1*, 34 for *ATXN2*, 73 for *ATXN3*, and 43 for *HTT*.

**Table 3:** Overview of the detection results for the known pathogenic variants sequenced with ONT long-read adaptive sampling

Single-nucleotide variants and Indels				
Sample ID	Known pathogenic variant	EPI2ME	NCRF	IGV
L-15936	*GBA1* c.1223C > T, p.T408M	Detected	-	-
L-15936	*LRRK2* c.6055G > A, p.G2019S	Detected	-	-
iPS-L-3244	*PRKN* c.823C > T, p.R275W	Detected	-	-
SFC817	*PRKN* c.971del, p.V324Afs*111	Detected	-	-
L-13754	*PRKN* c.101_102del, p.Q34Rfs*5	Detected	-	-
L-26618	*RAB32* c.213C > G, p.S71R	Detected	-	-
Structural variants				
Sample ID	Known pathogenic variant	EPI2ME	NCRF	IGV
SFC817	*PRKN* ex7del	Detected	-	-
iPS-L-3244	*PRKN* ex1del	Not detected	-	Observed
L-13754	*PRKN* ex2-3del	Not detected	-	Observed
iPS-L-3034	*PRKN* ex2del	Not detected	-	Observed
iPS-L-3034	*PRKN* ex3-5del	Detected	-	-
SFC831	*SNCA* triplication	Not detected	-	Not observed
Repeat expansions				
Sample ID	Known pathogenic variant	EPI2ME	NCRF	IGV
L-20456	*ATXN1* repeat expansion	Detected	-	-
L-20468	*ATXN2* repeat expansion	Detected	-	-
L-10180	*ATXN3* repeat expansion	Detected	-	-
L-20677	*HTT* repeat expansion	Detected	-	-
L-1689	*DAB1* repeat expansion	Not detected	Detected	-
L-15764	*FGF14* repeat expansion	Not detected	Detected	-
L-20799	*C9orf72* repeat expansion	Inconclusive	Not detected	Inconclusive
L-14372	*TAF1*-SVA repeat expansion	Not detected	Not detected	Inconclusive

D298, L-4474, and L-8319 are not listed as these individuals were idiopathic and had no known pathogenic variants.

*EPI2ME* Nanopore human variation workflow, *NCRF* Noise Cancelling Repeat Finder, *IGV* Integrative Genomics viewer, “-” The method was not necessary to employ after the detection of the variant by either EPI2ME or NCRF.

Currently, EPI2ME uses an input file of known repeat expansion loci, repeat motifs, pathogenic repeat lengths, and associated repeat expansion disorders, which does not yet include the genes *FGF14*, *DAB1*, and *TAF1*. Thus, Straglr could not detect the pathogenic repeat expansions in *DAB1*, *FGF14*, and the *TAF1*-SVA found in L-1689, L-15764, and L-14372, respectively (Table 3). The standalone version of Straglr, using a custom input BED file, detected a pathogenic *DAB1* (ATTTC)_123_ repeat expansion for L-1689 based on two reads (original RN = 116) and a pathogenic *FGF14* (GAA)_350_ repeat expansion for L-15764 based on 31 reads (original RN = 330). The *TAF1*-SVA repeat expansion was not detected.

Prior analysis of L-20799 using Repeat-primed PCR revealed an expansion in the *C9orf72* locus, but no exact RN was determined. Out of 41 reads covering *C9orf72* in L-20799, the workflow version of Straglr identified six expanded reads with an RN between 482 and 1660, but called the expansion ultimately as benign.

### Detection of the known pathogenic variants with Noise Cancelling Repeat Finder

Noise Cancelling Repeat Finder (NCRF), a short tandem repeat alignment tool, confirmed the repeat expansions in *ATXN1* (RN = 42)*, ATXN2* (RN = 34)*, ATXN3* (RN = 70), and *HTT* (RN = 41) detected by Straglr with a slight deviation of 3-4 repeat units. It also detected the pathogenic *DAB1* (ATTTC)_105_ repeat expansion for L-1689 (Supplementary Fig. 1) and the pathogenic *FGF14* (GAA)_349_ repeat expansion for L-15764 (Supplementary Fig. 2), with a bigger deviation of 18 repeat units for *DAB1* compared to the Straglr result. While NCRF detected 13 reads with high repeat lengths (2000–10,000 bp) in *C9orf72*, no exact repeat number could be determined due to the high variability (Supplementary Fig. 3). The expected repeat expansion in *TAF1*-SVA could not be detected (Table 3).

### Evaluation of the variants in the Integrative Genomics Viewer

To thoroughly assess the detection limit of our adaptive sampling panel, the Integrative Genomics Viewer (IGV) was used to visually inspect six known pathogenic variants that EPI2ME and NCRF did not detect. The presence of a 20 kb deletion spanning exon 1 of *PRKN* was identified in the region chr6:162,710,190-162,729,371 for iPS-L-3244 (Supplementary Fig. 4). A total of 20 reads of haplotype 1 (red) mapped to the deleted region. A single read of haplotype 2 (blue) carried the left breakpoint of the deletion as highlighted by a stretch of mismatched bases (Supplementary Fig. 4A). The *PRKN* SNV c.823C > T was located on the other allele, i.e., haplotype 1 (red) (Supplementary Fig. 4B).

A 320 kb deletion spanning exons 2–3 of *PRKN* was identified in the region chr6:162,232,182-162,555,779 for L-13754 (Supplementary Fig. 5). Compared to the surrounding coverage of approximately 38X, a drop to 21X was observed in the deleted region, with mostly reads of haplotype 1 (red) remaining (Supplementary Fig. 5A). The small *PRKN* deletion c.101_102del was located in the deleted stretch on haplotype 1 (red) (Supplementary Fig. 5B).

Both a 250 kb deletion in the region chr6:162,029,818-162,280,411 spanning exon 3–5 of *PRKN*, and a 112 kb deletion in the region chr6:162,336,433-162,448,673 spanning exon 2 of *PRKN*, were visually confirmed in iPS-L-3034 (Supplementary Fig. 6). Compared to the surrounding coverage of 26X, the deleted regions showed a coverage of 14X and 10X, respectively. Inspection of the breakpoint regions revealed that the 250 kb deletion is located on haplotype 1 (red) (Supplementary Fig. 6A, B), while the other deletion is present on haplotype 2 (blue) (Supplementary Fig. 6C). Thus, all *PRKN* variants were confirmed to be compound heterozygous.

**Fig. 4:**
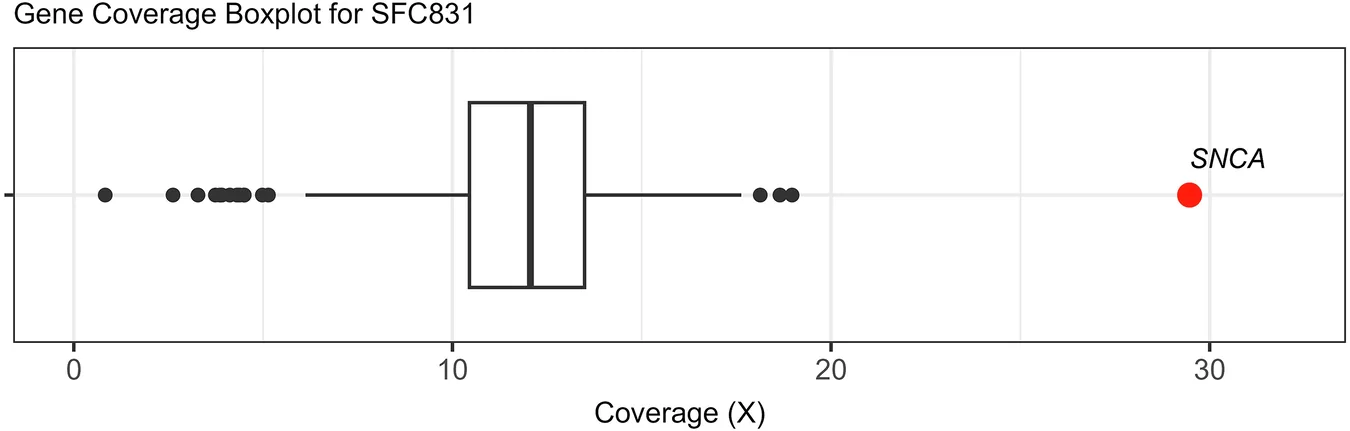
*SNCA* coverage in SFC831. The coverage of all 563 gene loci (mtDNA not considered) for the sample SFC831 is depicted in a box plot. A much deeper coverage (29.5X) was obtained for *SNCA* compared to all other genes, indicating the presence of the expected triplication spanning *SNCA*.

The presence of the 1.7 Mb triplication spanning the whole *SNCA* gene in SFC831 was previously validated using both long-read whole-genome sequencing and optical genome mapping^26^. While the variant could not be visually identified in the IGV reads, there was a 2.5-fold increase in *SNCA* coverage as compared to the mean coverage of all other gene loci in SFC831 (Fig. 4). In line with the EPI2ME results by Straglr, six out of the 41 reads covering *C9orf72* in L-20799 showed expansions ranging from 2877 to 9944 bp (Supplementary Fig. 7). At the position of the *TAF1* insertion in L-14372, three out of the seven on-target reads carried an insertion of approximately 2.7 kb (Supplementary Fig. 8). The low number of reads with a repeat expansion and the difference in repeat length make a reliable and accurate detection difficult. Therefore, both the *C9orf72* and *TAF1*-SVA repeat expansions were categorized as inconclusive. Seventeen out of the 20 known pathogenic variants across 15 individuals were clearly identified using the gene panel, while three variants (one *SNCA* triplication and two large repeat expansions in *C9orf72* and *TAF1*-SVA) were not accurately detected. No pathogenic variants were detected in the three individuals included as negative controls.

### Detection of additional pathogenic variants

Filtering of the EPI2ME output yielded 14 additional candidate variants not previously reported for these individuals. While nine variants could not be validated (Supplementary Table 4), four potentially pathogenic SNVs and SVs and one repeat expansion of uncertain significance (Table 4) were validated using either Sanger sequencing (Supplementary Fig. 9) or PCR and Agarose gel electrophoresis (Supplementary Fig. 10). For L-13754, who carried compound heterozygous *PRKN* deletions, Clair3 detected a nonsynonymous SNV in *STXBP2* (c.1247-1G > C). The deep learning-based splice variant prediction tool, SpliceAI, predicted a splice acceptor loss with a Delta score of 0.99, and both the CADD score of 32 and the ACMG score of 16 categorized the variant as pathogenic. The *STXBP2* gene associated with familial hemophagocytic lymphohistiocytosis (fHLH)^27,28^. The heterozygous SNV was present in the Sanger Chromatogram of the *STXBP2* PCR product (Supplementary Fig. 9A).

**Table 4:** The EPI2ME human variation workflow identified four potentially pathogenic variants and one variant of unknown significance that had not been previously reported for these individuals

Known pathogenic variants				
Sample ID	Gene	GRCh38 position	Variant	Associated phenotype
L-13754	*PRKN*	chr6:162,443,378	c.101_102del	Early-Onset Parkinson’s Disease
	*PRKN*	chr6:162,229,153-162,547,507	320 kb deletion (exon 2-exon 3)	Early-Onset Parkinson’s Disease
L-1689	*DAB1*	chr1:57,367,043	repeat expansion (ATTTC)_105_	Spinocerebellar Ataxia 37
SFC817	*PRKN*	chr6:161,548,965	c.971del	Early-Onset Parkinson’s Disease
	*PRKN*	chr6:161,590,316-161,794,731	204 kb deletion (exon 7)	Early-Onset Parkinson’s Disease
iPS-L-3034	*PRKN*	chr6:162,335,133-162,449,511	112 kb deletion (exon 2)	Early-Onset Parkinson’s Disease
	*PRKN*	chr6:162,028,488-162,281,482	250 kb deletion (exon 3-exon 5)	Early-Onset Parkinson’s Disease
L-15764	*FGF14*	chr13:102,161,570	repeat expansion (GAA)_349_	Spinocerebellar Ataxia 27B
**Additionally detected genetic variants**				
Sample ID	Gene	GRCh38 position	Variant	Associated phenotype
L-13754	*STXBP2*	chr19:7,645,196	c.1247-1G > C	Familial Hemophagocytic Lymphohistiocytosis 5
L-1689	*AP4S1*	chr14:31,066,239	c.43C > T	Spastic Paraplegia 52
SFC817	*RARS2*	chr6:87,564,181	c.160_161del	Pontocerebellar Hypoplasia
iPS-L-3034	*ALS2*	chr2:201,703,123- 201,704,615	1.4 kb deletion (exon 32-intron 33)	Amyotrophic Lateral Sclerosis 2
L-15764	*CNBP*	chr3:129,172,624	repeat expansion (CCTG)_137_	Myotonic Dystrophy 2

These variants were validated using Sanger sequencing or PCR.

In L-1689, who carried a *DAB1* repeat expansion, a nonsynonymous SNV was identified in *AP4S1* (c.43C > T). A high CADD score of 39 and an ACMG score of 14 classified the variant as pathogenic, which is known to be associated with spastic paraplegia 52 (SPG52)^29^. The heterozygous SNV was present in the Sanger Chromatogram of the *AP4S1* PCR product (Supplementary Fig. 9B).

A small indel affecting *RARS2* (c.160_161delAA) was detected in SFC817, who carried compound heterozygous *PRKN* variants. As a frameshift indel, it was predicted to result in a complete loss of function. Pathogenic variants in *RARS2* are associated with pontocerebellar hypoplasia type 6 (PCH6)^30^. The heterozygous indel was present in the Sanger chromatogram of the *RARS2* PCR product (Supplementary Fig. 9C).

Sniffles2 called a 1.4 kb deletion spanning exon 32 to intron 33 of *ALS2* in iPS-L-3034, who carried compound heterozygous *PRKN* deletions. The *ALS2* deletion had a CADD score of 11.16 and an ACMG score of 0.9. Biallelic variants, including deletions, in *ALS2* are associated with juvenile amyotrophic lateral sclerosis 2^31^. Two discrete bands at the height of approximately 2.1 kb and 700 bp were identified for the *ALS2* PCR product (Supplementary Fig. 10A). The presence of the deletion was also validated in the native sample prior to iPSC reprogramming.

A repeat expansion of 137 repeat units (RU) with the motif CCTG was detected by Stranglr for one *CNBP* allele in L-15764, who carried an *FGF14* repeat expansion. NCRF also detected the variant with an RN of 140.8 (Supplementary Fig. 11). Repeat expansions over a threshold of 75 repeats with the TCTG and CCTG motifs are known to cause myotonic dystrophy 2 (DM2)^32,33^. Three discrete bands at the height of approximately 1.5 kb, 900 bp, and 700 bp were identified for the *CNBP* PCR product (Supplementary Fig. 10B). Thus, five patients with pathogenic variants associated with early-onset PD or spinocerebellar ataxia (SCA) also carried variants in genes linked to other neurodegenerative diseases or conditions, such as immune deficiency, but without corresponding clinical phenotypes, indicating that these represent incidental findings requiring cautious interpretation rather than established additional diagnoses. Interestingly, 13 patients also carried benign (AAAAG)_n_ expansions in *RFC1*, with repeat numbers ranging from 57 to 144 (Supplementary Table 5), below the pathogenic threshold of 250 for *RFC1*^34^.

## Discussion

In this study, we developed and evaluated a long-read adaptive sampling gene panel consisting of 564 gene loci related to PD, repeat expansion disorders, and other neurodegenerative movement disorders and assessed its technical feasibility and potential diagnostic utility in 18 individuals with prior genetic testing, including 15 positive controls and 3 negative disease controls.

By focusing on relevant genes, we achieved a mean coverage of >20X, comparable to whole-genome sequencing (WGS) for a single sample on the PromethION, with three samples multiplexed per flow cell. Our targeted long-read sequencing approach thus allows for cost reduction and lower data storage and transfer requirements. This is sufficient for the robust detection of SNVs, small Indels, and SVs^35^. An adjustment would be to reduce the multiplexing to 2 samples to achieve a mean coverage of >30X, thereby further optimizing the detection of repeat expansions^36^. The coverage over the different gene loci showed high variance, which could be attributed in part to some genes being located in hard-to-map regions. The genes *VARS1*, *VARS2*, *TRIM40*, and *NEU1* are all located within or near the major histocompatibility complex (MHC) on chromosome 6, a highly polymorphic and complex region that can hinder accurate mapping^37^. Similarly, *HLA-DRB5* is located in the highly polymorphic HLA class II region^38^. Twenty out of the 31 genes with a mean coverage <15X are also X-linked (e.g., *TAF1*) and thus less abundant in the ten male individuals of the cohort, while two genes on chr5, *OCLN* and *SLC6A3*, are known challenging medically relevant genes (CMRGs)^39^ that are hard to characterize using NGS. Software frameworks like BOSS-RUNS^40^, based on readfish^41^, would allow for dynamic adjustments during adaptive sampling to, for example, favor hard-to-map regions and achieve a more continuous coverage over all gene loci. However, in this case, as large copy number variants were assessed, the underlying coverage information needed to be preserved^40,42^. To this end, we selected the built-in adaptive sampling solution of the ONT operating software, MinKNOW, to benefit from active support and future performance improvements.

The mean on-target read N50 of 21.5 kb was reported to be suitable for the detection of SVs^43^, but it also showed high variance (SD = ± 11.3 kb), which may be attributed to samples originating from different countries and varying storage durations and conditions. In particular, L-20456, L-20468, and L-14372 showed high fragmentation (on-target read N50 < 6 kb). The iPSC samples, on the other hand, generally showed high on-target read N50 values (>30 kb) as they were freshly generated and processed for this study, comparable to more recently collected in-house samples like L-15764 (Supplementary Table 2).

Using the EPI2ME human variation workflow, 12 of the 20 expected pathogenic variants were directly called (60%; Clopper-Pearson 95% CI: 36%–81%), while the presence of five additional variants was verified using either NCRF, CuteSV, or manual inspection in IGV. This includes three initially missed *PRKN* SVs: two were not called due to SV caller constraints, and one was missed due to the limited buffer sequence of 50 kb. The other two were repeat expansions that were likely not directly called due to limited read length and coverage, and to a limitation of the GRCh38 reference regarding the *TAF1*-SVA insertion. In total, 17/20 (85%; Clopper–Pearson 95% CI: 62%–97%) expected pathogenic variants were identified (15% missed; Clopper-Pearson 95% CI: 3%–38%).

All six known SNVs and small indels investigated in this study play a role in either early-onset PD (*PRKN*)^44^, late-onset PD (*LRRK2*, *RAB32*)^45,46^, or are associated with increased PD risk (*GBA1* p.T408M)^47^. Their detection was especially successful, as all expected variants were identified. The base-calling accuracy of ONT has improved significantly over the years, with *Q*-scores exceeding 20 now being routinely achievable using R10 flow cell chemistry and super-accurate base calling^25,48^. This advancement enabled the robust analysis of small variants using Clair3 on our targeted long-read data. Nevertheless, further improvements are still warranted, as the accuracy in SNV detection within complex regions remains limited compared to NGS^49^. All of the six known pathogenic SVs investigated in this study, i.e., the *PRKN* deletions and *SNCA* triplication, are known to cause early-onset PD^44,50^. The current generation of SV-callers generally shows comparable performance^51^, yet all have struggled to detect large (>50 kb) SVs in the past^52^. Recent improvements in Sniffles2 version 2.5 enhanced the detection of large (>50 kb) variants^53^. Using default parameters, Sniffles2 correctly identified two *PRKN* deletions of 200 and 250 kb in SFC817 and iPS-L-3034. However, using a manually adjusted size parameter, *SVIM* showed the same performance, whereas CuteSV additionally detected the missed *PRKN* deletions of 112 and 320 kb. This highlights that there is still no universal gold standard for SV calling of long-read data that does not require manual adjustment, which complicates automation. The universally missed 20 kb *PRKN* deletion in iPS-L-3244 spanned exon 1 and was located on the boundary of the sequenced region of interest, potentially preventing its detection. While flanking sequences of 50 kb were previously successful in similar studies^24,54^, we recommend to increase the flanking sequence for regions with a higher likelihood of SV formation, e.g., *PRKN*, located in a common fragile site^55,56^, or in general for genes known to be challenging to characterize due to their position in complex or highly repetitive regions (Supplementary Table 1). EPI2ME also includes the read depth based variant caller Spectre, which is optimized for CNVs ≥100 kb and could likely identify the large *PRKN* deletions in WGS data. However, it is not designed for targeted sequencing data and was therefore not applicable. While haplotagged reads enabled the detection and phasing of all *PRKN* variants in IGV, there remains potential to optimize the detection of large SVs, as manual inspection (~30 min per sample) might not be feasible in every analysis setting. Similarly, the *SNCA* triplication in SFC831 was not detected by any SV-caller, as the original variant spanned 1.7 Mb and increased the coverage of the entire *SNCA* locus. This emphasizes the need for specialized tools to detect copy number changes in targeted sequencing data^57^. The data generated here could serve as a benchmark for advancing these approaches.

The eight known pathogenic repeat expansions in this study cause different types of spinocerebellar ataxia (*ATXN1*, *ATXN2*, *ATXN3*, *DAB1*, *FGF14*)^58–62^, Huntington’s disease (*HTT*)^63^, amyotrophic lateral sclerosis (ALS) or FTD (*C9orf72*)^64,65^, and X-linked dystonia-parkinsonism (*XDP*-SVA)^66^. EPI2ME and NCRF showed concordance for the RN of four repeat expansions, while the pathogenic repeat expansions in *DAB1* and *FGF14* were only detected by the standalone version of Straglr and NCRF. The deviation of 18 repeat units observed in the *DAB1* repeat expansion is likely due to different custom references. We expect a more standardized workflow to emerge as new findings (i.e., new loci and repeat motifs) are added to the reference list of Straglr in the workflow. The repeat expansion in *C9orf72* was inconclusive. Straglr detected six reads carrying a repeat expansion in *C9orf72*, which did not suffice to categorize it as pathogenic. NCRF also detected expanded reads; however, the varying repeat lengths prevented the determination of a reliable repeat number. The very long GC-rich repeats of the *C9orf72* repeat expansion, which can also form G-quadruplex structures^67^, require deep coverage to be characterized beyond the repeat number^68–70^. A recent study on the utility of adaptive sampling in the diagnosis of cerebellar ataxia also presents the read depth as a limitation of adaptive sampling and suggests a coverage of greater than 33X for the interpretation of repeat expansions in a diagnostic setting^36^. While we achieved a coverage of 43X over the *C9orf72* locus, it was likely still insufficient, as the obtained read N50 of 11 kb (Supplementary Table 3) was low. This case emphasizes the current limitations in studying very large repeat expansions. There is a trade-off between improved enrichment from a smaller targeted genome fraction and pore blocking from increased strand rejection^42^. However, in a diagnostic-focused setting, it may be warranted to reduce the number of target regions (Supplementary Table 1) to further optimize coverage, while remaining in the advised 1%–5% fraction of the genome, e.g., by removing the GWAS loci that are primarily relevant for research. Similar problems also arose in detecting the repeat expansion in *TAF1*-SVA, which was a special case because it is located within a SINE-VNTR-Alu (SVA) retrotransposon insertion in the gene *TAF1*^66,71^. This SVA retrotransposon is absent in the GRCh38 human reference genome, which likely led to reduced coverage as some reads of interest were falsely discarded as off-target during adaptive sampling. Additionally, the *TAF1* gene is located on the X chromosome, resulting in lower coverage in men. Therefore, we obtained only three on-target reads with the SVA retrotransposon in the *TAF1* gene, which was insufficient for NCRF to accurately determine the RN.

Using our adaptive sampling panel, we identified four additional potentially pathogenic variants and one variant of uncertain significance across all three tested types (SNVs, SVs, and repeat expansions), demonstrating the broad utility compared to more specialized diagnostic tests. The three SNVs in *STXBP2*, *AP4S1*, and *RARS2*, as well as the 1.4 kb deletion in *ALS2*, were heterozygous, and no additional SNVs or SVs on the other allele were identified. All four patients showed no signs of comorbidity consistent with either fHLH, SPG52, PCH6, or ALS2, which is in line with our expectations, as these conditions exhibit autosomal recessive inheritance. DM2 has an autosomal dominant inheritance pattern, with a (CCTG)_137_ repeat expansion in *CNBP* being considered pathogenic^53,54^. However, no myotonic signs were observed, and all clinical signs are consistent with the initial diagnosis of autosomal dominant spinocerebellar ataxia 27 (SCA27), which is caused by the validated repeat expansion in *FGF14*^58,72^. A further complication is the presence of unspecific bands in the Agarose gel of the *CNBP* PCR validation. In addition to the expected band of the PCR product carrying the *CNBP* repeat expansion at approximately 700 bp, two additional bands are present at 900 bp and 1.5 kb. Furthermore, the median age of symptom onset for DM2 is 48 years^73^, and the patient was last examined at 73 years of age, underscoring the need for cautious interpretation and genetic counseling when incidental findings lack a clear clinical correlate. Further validation of the *CNBP* repeat expansion using an orthogonal method would be advised.

Our ONT adaptive sampling gene panel demonstrated strong detection capacities across all tested variant types, including phased large SVs and long repeat expansions, highlighting its potential as a cost-effective approach to leverage the advantages of long-read sequencing for clinical diagnosis in the future. A study focused on the diagnosis of spastic-ataxia disorders further emphasizes the method’s technical potential, as another ONT adaptive sampling panel detected pathogenic repeat expansions in 9/23 (39%) individuals with prior negative genetic test results^23^. Recent studies also present other specialized applications for adaptive sampling, e.g., to specifically validate CNVs^74^ or complex rearrangements^57^ in the clinic.

A possible use case of our neurology-focused panel in the context of genetic testing would be for an experienced clinician to recommend customizations based on specific clinical indicators and to apply the adaptive sampling panel as a first-line method, i.e., as a combined approach (capturing phased SNVs, large SVs and repeat expansions) for neurological disorders, or as a second-line method after a negative or inconclusive NGS sequencing result (Supplementary Fig. 12).

Under the research conditions described here, running the panel for three individuals multiplexed on a single flow cell costs the same as an ONT WGS run for a single individual (approximately 330–830 € per patient sample). Depending on the target query (e.g., large repeat expansions), multiplexing only two individuals would significantly increase coverage while remaining more cost-efficient than ONT WGS (approximately 500–1250 € per patient sample). It is still warranted to validate both pathogenic and inconclusive variants using methods such as PCR or Sanger sequencing, increasing total costs to approximately 500–1000 € per patient sample as a first-line test or to 600-1700 € as a second-line test (Supplementary Fig. 12). The costs for each genetic test (per sample with ~20-30X Coverage) were estimated based on international studies^75–77^, and on Chapter 11.4 of the EBM (German Uniform Evaluation Standard) catalog for the second quarter of 2026, provided by the National Association of Statutory Health Insurance Physicians (NASHIP)^78^. The long-read sequencing costs are based on self-estimates for research purposes. In clinical practice, however, this would currently be an underestimation, as additional factors (e.g., genetic counseling) contribute to the total cost.

A significant advantage is that the panel can be flexibly adapted by simply changing the text in the provided BED file. As newly discovered genes, risk loci^79,80^, and repeat motifs implicated in neurodegenerative movement disorder emerge, the panel should be updated and customized. The wide variety of targeted genes included in our study accounts for combined or atypical phenotypes^81^ and addresses limitations of a solely phenotype-driven analysis, as often many different genes can cause a given clinical phenotype^82^. At the same time, the panel can serve as a more accessible entry for non-clinicians to perform a combined assessment of SNVs, SVs, and repeat expansions.

There are limitations to address. This study demonstrates the technical feasibility of our adaptive sampling gene panel; however, a larger cohort would strengthen this potential, while a separate cohort of patients without prior knowledge of pathogenic variants would be needed to make broader claims about its performance in a genetic diagnostic setting. For four patients, genomic DNA was isolated from iPSC lines, as no blood was available. This allowed us to compare results from both biomaterials and was a practical approach to investigate the variants in this technical proof, but it would not be suitable for clinical diagnostics, as somatic variants might be introduced during cell reprogramming. Roadblocks, such as the need for extensive supporting infrastructure e.g., extraction and shearing of high-molecular-weight DNA^83^, limited bioinformatic resources (e.g., technology-matched variant catalogs and gold-standard variant callers)^84^, and a high concentration of DNA^36^, which currently limit the acceptance of long-read sequencing in clinical testing, also generally apply to our panel. As our data indicate, it is also currently advisable to manually inspect potential pathogenic variants, further increasing the need for specialized expertise and time. Finally, the obtained mean coverage of approximately 20X was sufficient for detecting large SVs but was likely too low to allow in-depth characterization of large repeat expansions. Most long-read software tools, especially variant callers, are currently optimized for WGS data, which may affect their performance when applied indiscriminately to adaptive sampling data, as highlighted for the CNV caller Spectre. We believe that as more specialized tools and settings for targeted long-read sequencing data become available, the advantages of performing a single comprehensive test will outweigh current concerns. To this end, the standalone EPI2ME software would likely be the solution of choice for improving automation compared to the manual command-line approach presented here. Further validation in larger and more heterogeneous patient cohorts will provide stronger support for the panel’s clinical potential.

## Methods

### Gene panel design

A novel gene panel focused on PD and repeat expansion disorders was designed using genes selected from established NGS-based genetic diagnostic panels for neurodegeneration offered by the Center for Genomics and Transcriptomics (CeGaT)^85^, the International Parkinson and Movement Disorder Society (MDS) Taskforce^86^, 90 PD genome-wide association study (GWAS) loci^87^, and known genes associated with repeat expansion disorders. In total, the panel consists of 564 target regions generated from the position of each gene locus ± 50 kb and the mitochondrial genome (Supplementary Table 1). The size distribution of the target regions and the total numbers for each chromosome are summarized in the supplementary materials (Supplementary Figs. 13 and 14).

### Patient demographics

Eighteen individuals from established patient cohorts with prior genetic testing were sequenced using adaptive sampling from ONT. Fifteen individuals were selected as positive controls based on their known pathogenic variants, and two individuals with idiopathic PD, as well as one individual with idiopathic frontotemporal dementia (FTD), were included as negative controls. Individuals carrying known pathogenic SVs or repeat expansions were also selected for investigation, as those are more difficult to identify with short-read sequencing. Patient demographics are provided in Table 2. The *RAB32* c.213C > G single-nucleotide variant (SNV) in L-26618^88^, four large SVs spanning *PRKN* and *SNCA* in iPS-L-3244, iPS-L-3034, and SFC831^26^, as well as the *DAB1* repeat expansion in L-1689^89^ and the *FGF14* repeat expansion in L-15764^58,90,91^ were previously described.

### Generation of induced pluripotent stem cells (iPSC)

Four skin fibroblast-derived induced pluripotent stem cell (iPSC) lines were examined in this study, as no whole blood was available for the patients. All iPSC lines were generated by overexpressing OCT4, SOX2, KLF4, and cMYC using Sendai virus to infect the fibroblast cultures, according to the manufacturer’s protocol (CytoTune Reprogramming Kit; Thermo Fisher Scientific). iPSC lines SFC831, iPS-L-3244, and iPS-L-3034 have been reported previously^26^, whereas iPSC line SFC817 has been newly generated. The clearance of CytoTune Sendai virus-delivered reprogramming genes has been confirmed for all lines (data not shown). The iPSCs were cultured in mTeSR1 medium (StemCell Technologies) on Matrigel-coated plates (BD Bioscience).

### ONT long-read sequencing

Genomic DNA was isolated from whole blood or iPSCs using automated DNA precipitation with the FlexiGene DNA Whole Blood Kit (Autogen), spin column purification with the High Pure Viral Nucleic Acid Kit (Roche), manual DNA precipitation, or with the Bionano Prep SP-G2 Blood & Cell Culture DNA Isolation Kit (Bionano) as previously described^26^.

Controlled shearing of the DNA was performed using the Megaruptor 3 from Diagenode, followed by size selection with the BluePippin from Sage Science, aiming at fragments of ≥30 kb^92^. The DNA quantity was monitored after each step using the Qubit fluorometer, and the fragment size distribution was assessed using either the Femto Pulse System or the Tapestation 4200 from Agilent Technologies, before and after shearing and size selection.

Library preparation was conducted according to an adapted version of the “1D Native barcoding genomic DNA Protocol” using the Native 96 Barcoding Kit (EXP-NBD114-96). This included ligation of the Barcodes to the ends of the DNA fragments and ligation and cleaning of the sequencing adaptors. Three samples were sequenced per R10.4.1 flow cell (FLO-PRO114M) on the PromethION from Oxford Nanopore Technologies (ONT) for 72 h, respectively (Supplementary Fig. 15).

Adaptive sampling (using the built-in Read until API) was activated in the device’s operating software, MinKNOW (version 24.06.15) to specifically enrich the regions of all gene loci in the panel, which were provided in a BED file containing the GRCh38 coordinates (Supplementary Table 1). Flanking regions of ±50 kb were included for each target gene locus to avoid coverage loss at the edges. All regions together correspond to a total length of 113,562,275 bp or 3.44% of the GRCh38 reference sequence. A fraction between 1% and 5% is recommended for optimal enrichment with adaptive sampling^42^.

### Bioinformatic analysis

The POD5 files were demultiplexed, and Dorado base-calling (version 0.5.1) was performed using the super accuracy (SUP) model (dna_r10.4.1_e8.2_400bps_sup@v4.3.0). Samtools (version 1.15)^93^ enabled the conversion and handling of FASTQ and BAM files. Unmapped BAM files served as input for the human variation workflow from the ONT bioinformatics software collection EPI2ME (version 2.7.0). The human variation workflow uses Nextflow (version 24.04.4)^94^ to integrate a collection of bioinformatic tools and establish reproducible and efficient conditions for the detection of genetic variants. This includes alignment to the GRCh38 genome reference using Minimap2 (version 2.24)^95^, SNV and indel calling using Clair3 (version 1.0.8)^96^, SV calling using Sniffles2 (version 2.6.2)^97^, and repeat expansion genotyping using Straglr (version 1.4.5)^98^. WhatsHap (version 2.0)^99^ performed phasing of the reads and identified variants. Sniffles2 was applied using default parameters (–long-del-length: 50000, –long-del-coverage: 0.66). The detection of pathogenic repeat expansions by Straglr relies on an input BED file of known repeat expansion loci, which currently excludes the genes *FGF14*, *DAB1*, and *TAF1* in the workflow. After the initial analysis, the standalone version of Straglr was applied to the GRCh38-aligned CRAM file using a custom BED file containing *FGF14*, *DAB1*, and *TAF1*.

The data were visualized with R (version 4.4.0)^100^ and the packages *tidyverse* (v2.0.0)^101^, *dplyr* (v1.1.4)^102^, *ggplot2* (v3.5.2)^103^, and *data*.table (v1.17.2)^104^. SnpEff^105^ and ClinVar^106^ provided annotations for the SNVs and indels. The SVs were annotated outside of EPI2ME using SVAFotate^107^ and AnnotSV (version 3.4.2)^108^.

Subsequent filtering of the variants was performed in R, focusing on rare variants (total genome and exome population allele frequency ≤1% in gnomAD 4.1.0) or those classified as “pathogenic” and “likely pathogenic” according to the ACMG guidelines^109^. The number of filtered candidates was further reduced by removing variants detected in multiple samples and STRs with benign motifs or repeat lengths well below the pathogenic threshold.

A workflow based on the short tandem repeat alignment tool, Noise Cancelling Repeat Finder (NCRF) (version 1.01.02)^110^, was used to detect repeat expansions that were not included or missed by the EPI2ME human variation workflow. Minimap2 (version 2.22) was applied to align the unmapped BAM files to the corresponding reference sequence of the gene, which was modified to include an expanded tract of the repeat motif. The SAM/BAM files were again handled with Samtools. The identification of repeat motifs was performed by NCRF, with a minimum of 10 detected repeat units. A maximum noise of 80% was applied as a filter, as previously tested for *FGF14* repeat expansions^90^. The resulting output was analyzed in R to calculate the number of repeats of the pathogenic allele. The detailed commands used in this study are provided on GitHub (https://github.com/aFienemann/Adaptive-sampling-gene-panel-for-PD-and-REDs). After all annotation and filtering steps, only the barcodes without context for the expected variants were provided to reduce confirmation bias during the analysis. The regions of all known pathogenic variants not detected using Sniffles2, Straglr, or NCRF were visualized using the Integrative Genomics Viewer (IGV)^111^. For this, the CRAM file containing the aligned reads produced by the EPI2ME human variation workflow was loaded into IGV using GRCh38 as the reference. SNVs and indels missed by the EPI2ME human variation workflow were additionally investigated by BCFtools (version 1.9)^112^, while missed SVs were further examined using both CuteSV (version 2.1.3)^113^ and SVIM (version 2.0)^114^. Parameters of both CuteSV (–max_size 350000) and SVIM (–max_sv_size 350000) were increased from the default value of 100,000 to allow for the detection of SVs up to 350 kb instead of 100 kb. As the cohort size was small, the Clopper-Pearson exact method was applied to obtain confidence intervals for the detection rate. Known variants that could be identified in the data but were not sufficiently characterized (e.g., repeat number) by our approach without prior knowledge were termed “inconclusive”. Using our filtering strategy, we detected additional potentially pathogenic variants besides the known pathogenic ones. These variants were validated using PCR-based Sanger sequencing for SNVs and indels (Supplementary Table 3) or agarose gel electrophoresis for SVs and repeat expansions.

### European Nucleotide Archive (ENA)

Mapped BAM files of all 15 positive controls with known pathogenic variants used in this study were deposited as a resource in the European Nucleotide Archive (ENA) at EMBL-EBI under accession number PRJEB105200 (https://www.ebi.ac.uk/ena/browser/view/PRJEB105200).

### Ethics approval and consent to participate

Written informed consent was obtained from all individuals and the study was conducted according to the guidelines of the Declaration of Helsinki, and approved by the Ethics Committees of the University of Lübeck, Germany (protocol code 16-039, date of approval 27 September 2019) and the P2N supervisory board, Kiel University, Germany (protocol code 2021-037, date of approval 16 September 2021).

## Supplementary information

Supplementary Information

## Data Availability

Mapped BAM files of all 15 positive controls with known pathogenic variants used in this study were deposited as a resource in the European Nucleotide Archive (ENA) at EMBL-EBI under accession number PRJEB105200 (https://www.ebi.ac.uk/ena/browser/view/PRJEB105200). The remaining datasets used and/or analyzed during the current study are available from the corresponding author on reasonable request.

## Code availability

The detailed commands used in this study are provided on GitHub (github.com/aFienemann/Adaptive-sampling-gene-panel-for-PD-and-REDs).
