# Supplementary material for: Unified long-read panel for Parkinson’s and repeat expansion disorders
Source: NPJ Parkinsons Dis. 2026 Sep 24;12:233. doi: 10.1038/s41531-026-01585-4 (PMC13612410; doi:10.1038/s41531-026-01585-4)
Supplement: Supplementary file 1 [file 41531_2026_1585_MOESM1_ESM.docx]

# Supplementary material

**Supplementary Table 1. Overview of all 564 gene loci included in the gene panel**.

| **Gene** | **Gene set** | **Chromosome** | **Start position (bp)** | **End position (bp)** | **Start (minus 50 kb buffer)** | **End (plus 50 kb buffer)** | **Size of target region (bp)** | **Comment** |
| --- | --- | --- | --- | --- | --- | --- | --- | --- |
| *AARS1* | CeGaT diagnostic panels (neurodegeneration) | chr16 | 70251983 | 70289707 | 70201983 | 70339707 | 137724 |  |
| *AARS2* | MDS Taskforce list (ataxia) | chr6 | 44298731 | 44313347 | 44248731 | 44363347 | 114616 |  |
| *ABCB7* | MDS Taskforce list (ataxia) | chrX | 75051048 | 75156732 | 75001048 | 75206732 | 205684 |  |
| *ABCD1* | MDS Taskforce list (ataxia) | chrX | 153724856 | 153744755 | 153674856 | 153794755 | 119899 |  |
| *ABHD12* | MDS Taskforce list (ataxia) | chr20 | 25294742 | 25390835 | 25244742 | 25440835 | 196093 |  |
| *ACAT1* | MDS Taskforce list (chorea) | chr11 | 108116695 | 108147603 | 108066695 | 108197603 | 130908 |  |
| *ACOX1* | CeGaT diagnostic panels (neurodegeneration) | chr17 | 75941507 | 75979177 | 75891507 | 76029177 | 137670 |  |
| *ADAR* | MDS Taskforce list (chorea) | chr1 | 154581695 | 154628013 | 154531695 | 154678013 | 146318 |  |
| *ADCY5* | CeGaT diagnostic panels (neurodegeneration) | chr3 | 123282296 | 123449090 | 123232296 | 123499090 | 266794 |  |
| *ADPRS* | MDS Taskforce list (ataxia) | chr1 | 36088892 | 36093932 | 36038892 | 36143932 | 105040 |  |
| *AFG3L2* | MDS Taskforce list (ataxia) | chr18 | 12328944 | 12377227 | 12278944 | 12427227 | 148283 |  |
| *AIMP1* | CeGaT diagnostic panels (neurodegeneration) | chr4 | 106315544 | 106349456 | 106265544 | 106399456 | 133912 |  |
| *AIMP2* | CeGaT diagnostic panels (neurodegeneration) | chr7 | 6009255 | 6023834 | 5959255 | 6073834 | 114579 | Twist Alliance Dark Genes |
| *ALDH18A1* | CeGaT diagnostic panels (neurodegeneration) | chr10 | 95605941 | 95656711 | 95555941 | 95706711 | 150770 |  |
| *ALDH3A2* | CeGaT diagnostic panels (neurodegeneration) | chr17 | 19648136 | 19685760 | 19598136 | 19735760 | 137624 |  |
| *ALS2* | CeGaT diagnostic panels (neurodegeneration) | chr2 | 201700267 | 201782112 | 201650267 | 201832112 | 181845 |  |
| *ANG* | MDS Taskforce list (ataxia) | chr14 | 20684177 | 20698971 | 20634177 | 20748971 | 114794 |  |
| *ANO10* | MDS Taskforce list (ataxia) | chr3 | 43354859 | 43691594 | 43304859 | 43741594 | 436735 |  |
| *ANO3* | MDS Taskforce list (dystonia) | chr11 | 26188842 | 26663289 | 26138842 | 26713289 | 574447 |  |
| *AOPEP* | CeGaT diagnostic panels (neurodegeneration) | chr9 | 94726604 | 95151793 | 94676604 | 95201793 | 525189 |  |
| *AP1M1* | CeGaT diagnostic panels (neurodegeneration) | chr19 | 16197854 | 16245906 | 16147854 | 16295906 | 148052 |  |
| *AP4B1* | CeGaT diagnostic panels (neurodegeneration) | chr1 | 113894194 | 113905712 | 113844194 | 113955712 | 111518 |  |
| *AP4E1* | CeGaT diagnostic panels (neurodegeneration) | chr15 | 50908672 | 51005895 | 50858672 | 51055895 | 197223 |  |
| *AP4M1* | CeGaT diagnostic panels (neurodegeneration) | chr7 | 100100858 | 100110345 | 100050858 | 100160345 | 109487 |  |
| *AP4S1* | CeGaT diagnostic panels (neurodegeneration) | chr14 | 31025106 | 31130996 | 30975106 | 31180996 | 205890 |  |
| *AP5Z1* | CeGaT diagnostic panels (neurodegeneration) | chr7 | 4775615 | 4794397 | 4725615 | 4844397 | 118782 |  |
| *APOE* | CeGaT diagnostic panels (neurodegeneration) | chr19 | 44905791 | 44909393 | 44855791 | 44959393 | 103602 |  |
| *APP* | CeGaT diagnostic panels (neurodegeneration) | chr21 | 25880535 | 26171128 | 25830535 | 26221128 | 390593 |  |
| *APTX* | MDS Taskforce list (ataxia) | chr9 | 32886601 | 33025130 | 32836601 | 33075130 | 238529 |  |
| *AR* | MDS Taskforce list (ataxia) | chrX | 67544021 | 67730619 | 67494021 | 67780619 | 286598 |  |
| *ARSA* | CeGaT diagnostic panels (neurodegeneration) | chr22 | 50622754 | 50628173 | 50572754 | 50678173 | 105419 |  |
| *ARX* | MDS Taskforce list (ataxia) | chrX | 25003694 | 25016420 | 24953694 | 25066420 | 112726 |  |
| *ASPA* | CeGaT diagnostic panels (neurodegeneration) | chr17 | 3472374 | 3503405 | 3422374 | 3553405 | 131031 |  |
| *ASXL3* | GWAS loci (PD) | chr18 | 33578219 | 33751195 | 33528219 | 33801195 | 272976 |  |
| *ATCAY* | CeGaT diagnostic panels (neurodegeneration) | chr19 | 3879864 | 3928082 | 3829864 | 3978082 | 148218 |  |
| *ATG14* | CeGaT diagnostic panels (neurodegeneration) | chr14 | 55366391 | 55411830 | 55316391 | 55461830 | 145439 |  |
| *ATL1* | CeGaT diagnostic panels (neurodegeneration) | chr14 | 50532509 | 50634017 | 50482509 | 50684017 | 201508 |  |
| *ATM* | MDS Taskforce list (ataxia) | chr11 | 108222804 | 108369102 | 108172804 | 108419102 | 246298 |  |
| *ATN1* | MDS Taskforce list (ataxia) | chr12 | 6924463 | 6942321 | 6874463 | 6992321 | 117858 |  |
| *ATP13A2* | CeGaT diagnostic panels (neurodegeneration) | chr1 | 16985958 | 17011928 | 16935958 | 17061928 | 125970 |  |
| *ATP1A2* | CeGaT diagnostic panels (neurodegeneration) | chr1 | 160115759 | 160143591 | 160065759 | 160193591 | 127832 |  |
| *ATP1A3* | MDS Taskforce list (dystonia) | chr19 | 41966582 | 41997497 | 41916582 | 42047497 | 130915 |  |
| *ATP6AP2* | CeGaT diagnostic panels (neurodegeneration) | chrX | 40579372 | 40606848 | 40529372 | 40656848 | 127476 |  |
| *ATP7B* | MDS Taskforce list (ataxia) | chr13 | 51930436 | 52012125 | 51880436 | 52062125 | 181689 |  |
| *ATP8A2* | CeGaT diagnostic panels (neurodegeneration) | chr13 | 25371974 | 26025851 | 25321974 | 26075851 | 753877 |  |
| *ATXN1* | MDS Taskforce list (ataxia) | chr6 | 16299112 | 16761491 | 16249112 | 16811491 | 562379 |  |
| *ATXN10* | MDS Taskforce list (ataxia) | chr22 | 45671798 | 45845307 | 45621798 | 45895307 | 273509 |  |
| *ATXN2* | MDS Taskforce list (ataxia) | chr12 | 111443485 | 111599676 | 111393485 | 111649676 | 256191 |  |
| *ATXN3* | MDS Taskforce list (ataxia) | chr14 | 92044496 | 92106621 | 91994496 | 92156621 | 162125 |  |
| *ATXN7* | MDS Taskforce list (ataxia) | chr3 | 63863155 | 64003462 | 63813155 | 64053462 | 240307 |  |
| *ATXN8OS* | Other repeat expansion loci | chr13 | 70107213 | 70171738 | 70057213 | 70221738 | 164525 |  |
| *B4GALNT1* | MDS Taskforce list (ataxia) | chr12 | 57623409 | 57633239 | 57573409 | 57683239 | 109830 |  |
| *BAG3* | GWAS loci (PD) | chr10 | 119651380 | 119677819 | 119601380 | 119727819 | 126439 |  |
| *BCAP31* | MDS Taskforce list (dystonia) | chrX | 153700492 | 153724565 | 153650492 | 153774565 | 124073 |  |
| *BEAN1* | MDS Taskforce list (ataxia) | chr16 | 66427295 | 66493529 | 66377295 | 66543529 | 166234 |  |
| *BICD2* | CeGaT diagnostic panels (neurodegeneration) | chr9 | 92711363 | 92764833 | 92661363 | 92814833 | 153470 |  |
| *BIN3* | GWAS loci (PD) | chr8 | 22620418 | 22669148 | 22570418 | 22719148 | 148730 |  |
| *BOLA3* | CeGaT diagnostic panels (neurodegeneration) | chr2 | 74135400 | 74147912 | 74085400 | 74197912 | 112512 |  |
| *BRIP1* | CeGaT diagnostic panels (neurodegeneration) | chr17 | 61679139 | 61863559 | 61629139 | 61913559 | 284420 |  |
| *BSCL2* | CeGaT diagnostic panels (neurodegeneration) | chr11 | 62689289 | 62709845 | 62639289 | 62759845 | 120556 |  |
| *BST1* | GWAS loci (PD) | chr4 | 15703065 | 15738313 | 15653065 | 15788313 | 135248 |  |
| *C19orf12* | MDS Taskforce list (dystonia) | chr19 | 29698937 | 29715789 | 29648937 | 29765789 | 116852 |  |
| *C5orf24* | GWAS loci (PD) | chr5 | 134845680 | 134859735 | 134795680 | 134909735 | 114055 |  |
| *C9orf72* | MDS Taskforce list (ataxia) | chr9 | 27535640 | 27573866 | 27485640 | 27623866 | 138226 |  |
| *CA2* | MDS Taskforce list (ataxia) | chr8 | 85463968 | 85481493 | 85413968 | 85531493 | 117525 |  |
| *CA8* | MDS Taskforce list (ataxia) | chr8 | 60185412 | 60281400 | 60135412 | 60331400 | 195988 |  |
| *CAB39L* | GWAS loci (PD) | chr13 | 49308650 | 49444064 | 49258650 | 49494064 | 235414 |  |
| *CACNA1A* | Other repeat expansion loci | chr19 | 13206442 | 13624489 | 13156442 | 13674489 | 518047 |  |
| *CACNA1G* | MDS Taskforce list (ataxia) | chr17 | 50560715 | 50627474 | 50510715 | 50677474 | 166759 |  |
| *CACNB4* | CeGaT diagnostic panels (neurodegeneration) | chr2 | 151832771 | 152099167 | 151782771 | 152149167 | 366396 |  |
| *CAMK2D* | GWAS loci (PD) | chr4 | 113418054 | 113761927 | 113368054 | 113811927 | 443873 |  |
| *CAMTA1* | CeGaT diagnostic panels (neurodegeneration) | chr1 | 6785454 | 7769706 | 6735454 | 7819706 | 1084252 |  |
| *CAPN1* | MDS Taskforce list (ataxia) | chr11 | 65180566 | 65212006 | 65130566 | 65262006 | 131440 |  |
| *CAPRIN2* | CeGaT diagnostic panels (neurodegeneration) | chr12 | 30709552 | 30823302 | 30659552 | 30873302 | 213750 |  |
| *CARS2* | CeGaT diagnostic panels (neurodegeneration) | chr13 | 110641412 | 110713603 | 110591412 | 110763603 | 172191 |  |
| *CASC16* | GWAS loci (PD) | chr16 | 52552084 | 52652132 | 52502084 | 52702132 | 200048 |  |
| *CCDC88C* | MDS Taskforce list (ataxia) | chr14 | 91271323 | 91417844 | 91221323 | 91467844 | 246521 |  |
| *CCN3* | CeGaT diagnostic panels (neurodegeneration) | chr8 | 119416446 | 119424434 | 119366446 | 119474434 | 107988 |  |
| *CD19* | GWAS loci (PD) | chr16 | 28931965 | 28939342 | 28881965 | 28989342 | 107377 |  |
| *CHCHD10* | CeGaT diagnostic panels (neurodegeneration) | chr22 | 23765834 | 23767972 | 23715834 | 23817972 | 102138 |  |
| *CHCHD2* | MDS Taskforce list (PD) | chr7 | 56101573 | 56106476 | 56051573 | 56156476 | 104903 |  |
| *CHD9* | GWAS loci (PD) | chr16 | 53054991 | 53329150 | 53004991 | 53379150 | 374159 |  |
| *CHMP2B* | CeGaT diagnostic panels (neurodegeneration) | chr3 | 87227271 | 87255556 | 87177271 | 87305556 | 128285 |  |
| *CHRNB1* | GWAS loci (PD) | chr17 | 7445061 | 7457710 | 7395061 | 7507710 | 112649 |  |
| *CIZ1* | CeGaT diagnostic panels (neurodegeneration) | chr9 | 128161251 | 128204383 | 128111251 | 128254383 | 143132 |  |
| *CLCN2* | MDS Taskforce list (ataxia) | chr3 | 184346185 | 184361650 | 184296185 | 184411650 | 115465 |  |
| *CLCN3* | GWAS loci (PD) | chr4 | 169612633 | 169723673 | 169562633 | 169773673 | 211040 |  |
| *CLN3* | CeGaT diagnostic panels (neurodegeneration) | chr16 | 28474111 | 28495575 | 28424111 | 28545575 | 121464 |  |
| *CLN5* | MDS Taskforce list (ataxia) | chr13 | 76990660 | 77019143 | 76940660 | 77069143 | 128483 |  |
| *CLN6* | MDS Taskforce list (ataxia) | chr15 | 68206992 | 68257211 | 68156992 | 68307211 | 150219 |  |
| *CLN8* | CeGaT diagnostic panels (neurodegeneration) | chr8 | 1755778 | 1801711 | 1705778 | 1851711 | 145933 |  |
| *CNBP* | Other repeat expansion loci | chr3 | 129167827 | 129183922 | 129117827 | 129233922 | 116095 |  |
| *COA7* | MDS Taskforce list (ataxia) | chr1 | 52684449 | 52698347 | 52634449 | 52748347 | 113898 |  |
| *COA8* | CeGaT diagnostic panels (neurodegeneration) | chr14 | 103562960 | 103607523 | 103512960 | 103657523 | 144563 |  |
| *COASY* | CeGaT diagnostic panels (neurodegeneration) | chr17 | 42561467 | 42566277 | 42511467 | 42616277 | 104810 |  |
| *COL4A1* | CeGaT diagnostic panels (neurodegeneration) | chr13 | 110148963 | 110307202 | 110098963 | 110357202 | 258239 |  |
| *COL4A2* | CeGaT diagnostic panels (neurodegeneration) | chr13 | 110305812 | 110513209 | 110255812 | 110563209 | 307397 |  |
| *COL6A3* | CeGaT diagnostic panels (neurodegeneration) | chr2 | 237324003 | 237414328 | 237274003 | 237464328 | 190325 |  |
| *COLGALT1* | CeGaT diagnostic panels (neurodegeneration) | chr19 | 17555649 | 17583162 | 17505649 | 17633162 | 127513 |  |
| *COQ8A* | CeGaT diagnostic panels (neurodegeneration) | chr1 | 226940286 | 226987544 | 226890286 | 227037544 | 147258 |  |
| *COX10* | MDS Taskforce list (ataxia) | chr17 | 14069490 | 14231736 | 14019490 | 14281736 | 262246 |  |
| *COX15* | MDS Taskforce list (ataxia) | chr10 | 99710868 | 99732127 | 99660868 | 99782127 | 121259 |  |
| *COX20* | MDS Taskforce list (ataxia) | chr1 | 244835616 | 244845057 | 244785616 | 244895057 | 109441 |  |
| *COX6B1* | CeGaT diagnostic panels (neurodegeneration) | chr19 | 35648323 | 35658782 | 35598323 | 35708782 | 110459 | Twist Alliance Dark Genes; Challenging medically relevant gene (CMRG) |
| *CP* | MDS Taskforce list (ataxia) | chr3 | 149162410 | 149221829 | 149112410 | 149271829 | 159419 |  |
| *CPT1C* | CeGaT diagnostic panels (neurodegeneration) | chr19 | 49690898 | 49713731 | 49640898 | 49763731 | 122833 |  |
| *CRHR1* | GWAS loci (PD) | chr17 | 45784277 | 45835828 | 45734277 | 45885828 | 151551 |  |
| *CRLS1* | GWAS loci (PD) | chr20 | 6006093 | 6040053 | 5956093 | 6090053 | 133960 |  |
| *CRY2* | CeGaT diagnostic panels (neurodegeneration) | chr11 | 45847118 | 45883248 | 45797118 | 45933248 | 136130 |  |
| *CSF1R* | CeGaT diagnostic panels (neurodegeneration) | chr5 | 150053291 | 150113372 | 150003291 | 150163372 | 160081 |  |
| *CSTB* | MDS Taskforce list (ataxia) | chr21 | 43772511 | 43776330 | 43722511 | 43826330 | 103819 |  |
| *CTBP1* | CeGaT diagnostic panels (neurodegeneration) | chr4 | 1211445 | 1250333 | 1161445 | 1300333 | 138888 |  |
| *CTC1* | MDS Taskforce list (ataxia) | chr17 | 8224815 | 8248058 | 8174815 | 8298058 | 123243 |  |
| *CTSA* | MDS Taskforce list (ataxia) | chr20 | 45890144 | 45898949 | 45840144 | 45948949 | 108805 | Twist Alliance Dark Genes |
| *CTSB* | GWAS loci (PD) | chr8 | 11842524 | 11869533 | 11792524 | 11919533 | 127009 |  |
| *CTSD* | CeGaT diagnostic panels (neurodegeneration) | chr11 | 1752752 | 1764573 | 1702752 | 1814573 | 111821 |  |
| *CTSF* | MDS Taskforce list (ataxia) | chr11 | 66563464 | 66568879 | 66513464 | 66618879 | 105415 |  |
| *CWF19L1* | MDS Taskforce list (ataxia) | chr10 | 100232298 | 100267680 | 100182298 | 100317680 | 135382 |  |
| *CYP27A1* | MDS Taskforce list (ataxia) | chr2 | 218781749 | 218815293 | 218731749 | 218865293 | 133544 |  |
| *CYP2U1* | CeGaT diagnostic panels (neurodegeneration) | chr4 | 107931549 | 107953461 | 107881549 | 108003461 | 121912 |  |
| *CYP7B1* | MDS Taskforce list (ataxia) | chr8 | 64587763 | 64798737 | 64537763 | 64848737 | 310974 |  |
| *DAB1* | MDS Taskforce list (ataxia) | chr1 | 56994778 | 58546734 | 56944778 | 58596734 | 1651956 |  |
| *DARS1* | CeGaT diagnostic panels (neurodegeneration) | chr2 | 135905881 | 135986100 | 135855881 | 136036100 | 180219 |  |
| *DARS2* | MDS Taskforce list (ataxia) | chr1 | 173824653 | 173858808 | 173774653 | 173908808 | 134155 |  |
| *DCAF17* | MDS Taskforce list (chorea) | chr2 | 171434217 | 171485052 | 171384217 | 171535052 | 150835 |  |
| *DCTN1* | MDS Taskforce list (PD) | chr2 | 74361154 | 74392087 | 74311154 | 74442087 | 130933 |  |
| *DDC* | MDS Taskforce list (dystonia) | chr7 | 50458436 | 50565405 | 50408436 | 50615405 | 206969 |  |
| *DDHD1* | CeGaT diagnostic panels (neurodegeneration) | chr14 | 53036745 | 53153323 | 52986745 | 53203323 | 216578 |  |
| *DDHD2* | MDS Taskforce list (ataxia) | chr8 | 38225218 | 38275558 | 38175218 | 38325558 | 150340 |  |
| *DLG2* | GWAS loci (PD) | chr11 | 83455012 | 85628335 | 83405012 | 85678335 | 2273323 |  |
| *DMPK* | Other repeat expansion loci | chr19 | 45769709 | 45782552 | 45719709 | 45832552 | 112843 | Twist Alliance Dark Genes; Challenging medically relevant gene (CMRG) |
| *DNAH17* | GWAS loci (PD) | chr17 | 78423697 | 78577396 | 78373697 | 78627396 | 253699 |  |
| *DNAJC12* | MDS Taskforce list (dystonia) | chr10 | 67796669 | 67838188 | 67746669 | 67888188 | 141519 |  |
| *DNAJC13* | CeGaT diagnostic panels (neurodegeneration) | chr3 | 132417502 | 132539032 | 132367502 | 132589032 | 221530 |  |
| *DNAJC5* | CeGaT diagnostic panels (neurodegeneration) | chr20 | 63895126 | 63936031 | 63845126 | 63986031 | 140905 |  |
| *DNAJC6* | MDS Taskforce list (dystonia) | chr1 | 65248219 | 65415871 | 65198219 | 65465871 | 267652 |  |
| *DNMT1* | MDS Taskforce list (ataxia) | chr19 | 10133342 | 10231286 | 10083342 | 10281286 | 197944 |  |
| *DSTYK* | CeGaT diagnostic panels (neurodegeneration) | chr1 | 205142505 | 205211702 | 205092505 | 205261702 | 169197 |  |
| *DYRK1A* | GWAS loci (PD) | chr21 | 37365573 | 37526358 | 37315573 | 37576358 | 260785 |  |
| *E2F1* | CeGaT diagnostic panels (neurodegeneration) | chr20 | 33675477 | 33686385 | 33625477 | 33736385 | 110908 |  |
| *EARS2* | CeGaT diagnostic panels (neurodegeneration) | chr16 | 23520754 | 23557731 | 23470754 | 23607731 | 136977 |  |
| *EIF2AK2* | MDS Taskforce list (dystonia) | chr2 | 37099210 | 37157522 | 37049210 | 37207522 | 158312 |  |
| *EIF2B1* | MDS Taskforce list (ataxia) | chr12 | 123620406 | 123633766 | 123570406 | 123683766 | 113360 |  |
| *EIF2B2* | MDS Taskforce list (ataxia) | chr14 | 75002921 | 75012366 | 74952921 | 75062366 | 109445 |  |
| *EIF2B3* | MDS Taskforce list (ataxia) | chr1 | 44850522 | 44986722 | 44800522 | 45036722 | 236200 |  |
| *EIF2B4* | MDS Taskforce list (ataxia) | chr2 | 27364352 | 27370338 | 27314352 | 27420338 | 105986 |  |
| *EIF2B5* | MDS Taskforce list (ataxia) | chr3 | 184135038 | 184146127 | 184085038 | 184196127 | 111089 | Twist Alliance Dark Genes; Challenging medically relevant gene (CMRG) |
| *EIF4A2* | CeGaT diagnostic panels (neurodegeneration) | chr3 | 186783205 | 186789897 | 186733205 | 186839897 | 106692 |  |
| *EIF4G1* | CeGaT diagnostic panels (neurodegeneration) | chr3 | 184314495 | 184335358 | 184264495 | 184385358 | 120863 |  |
| *ELOVL4* | MDS Taskforce list (ataxia) | chr6 | 79914814 | 79947553 | 79864814 | 79997553 | 132739 |  |
| *ELOVL5* | MDS Taskforce list (ataxia) | chr6 | 53267398 | 53349179 | 53217398 | 53399179 | 181781 |  |
| *ELOVL7* | GWAS loci (PD) | chr5 | 60751791 | 60844274 | 60701791 | 60894274 | 192483 |  |
| *EMC1* | CeGaT diagnostic panels (neurodegeneration) | chr1 | 19215660 | 19251552 | 19165660 | 19301552 | 135892 |  |
| *EP300* | CeGaT diagnostic panels (neurodegeneration) | chr22 | 41092510 | 41180077 | 41042510 | 41230077 | 187567 |  |
| *EPM2A* | MDS Taskforce list (ataxia) | chr6 | 145382535 | 145736023 | 145332535 | 145786023 | 453488 |  |
| *EPRS1* | CeGaT diagnostic panels (neurodegeneration) | chr1 | 219968600 | 220046530 | 219918600 | 220096530 | 177930 |  |
| *ERCC6* | CeGaT diagnostic panels (neurodegeneration) | chr10 | 49454168 | 49539538 | 49404168 | 49589538 | 185370 |  |
| *ERCC8* | CeGaT diagnostic panels (neurodegeneration) | chr5 | 60866454 | 60945073 | 60816454 | 60995073 | 178619 |  |
| *ERLIN1* | CeGaT diagnostic panels (neurodegeneration) | chr10 | 100150094 | 100186033 | 100100094 | 100236033 | 135939 |  |
| *ERLIN2* | CeGaT diagnostic panels (neurodegeneration) | chr8 | 37736601 | 37758422 | 37686601 | 37808422 | 121821 |  |
| *FA2H* | MDS Taskforce list (ataxia) | chr16 | 74712955 | 74774831 | 74662955 | 74824831 | 161876 |  |
| *FAM171A2* | CeGaT diagnostic panels (neurodegeneration) | chr17 | 44353215 | 44363853 | 44303215 | 44413853 | 110638 |  |
| *FAM47E* | GWAS loci (PD) | chr4 | 76214040 | 76283783 | 76164040 | 76333783 | 169743 |  |
| *FAM47E-STBD1* | GWAS loci (PD) | chr4 | 76251721 | 76311129 | 76201721 | 76361129 | 159408 |  |
| *FARS2* | CeGaT diagnostic panels (neurodegeneration) | chr6 | 5261044 | 5829192 | 5211044 | 5879192 | 668148 |  |
| *FASN* | CeGaT diagnostic panels (neurodegeneration) | chr17 | 82078338 | 82098294 | 82028338 | 82148294 | 119956 |  |
| *FBRSL1* | GWAS loci (PD) | chr12 | 132489551 | 132585188 | 132439551 | 132635188 | 195637 |  |
| *FBXO7* | MDS Taskforce list (PD) | chr22 | 32474676 | 32498829 | 32424676 | 32548829 | 124153 |  |
| *FCGR2A* | GWAS loci (PD) | chr1 | 161505430 | 161524013 | 161455430 | 161574013 | 118583 |  |
| *FGF14* | MDS Taskforce list (ataxia) | chr13 | 101710804 | 102402457 | 101660804 | 102452457 | 791653 |  |
| *FGF20* | GWAS loci (PD) | chr8 | 16992181 | 17002345 | 16942181 | 17052345 | 110164 |  |
| *FIG4* | CeGaT diagnostic panels (neurodegeneration) | chr6 | 109690609 | 109878098 | 109640609 | 109928098 | 287489 |  |
| *FKRP* | CeGaT diagnostic panels (neurodegeneration) | chr19 | 46746046 | 46776988 | 46696046 | 46826988 | 130942 |  |
| *FKTN* | CeGaT diagnostic panels (neurodegeneration) | chr9 | 105558122 | 105653820 | 105508122 | 105703820 | 195698 |  |
| *FLVCR1* | CeGaT diagnostic panels (neurodegeneration) | chr1 | 212858275 | 212899363 | 212808275 | 212949363 | 141088 |  |
| *FMR1* | Other repeat expansion loci | chrX | 147911919 | 147951125 | 147861919 | 148001125 | 139206 |  |
| *FOLR1* | MDS Taskforce list (ataxia) | chr11 | 72189558 | 72196323 | 72139558 | 72246323 | 106765 |  |
| *FOXC1* | CeGaT diagnostic panels (neurodegeneration) | chr6 | 1609915 | 1613897 | 1559915 | 1663897 | 103982 |  |
| *FOXRED1* | MDS Taskforce list (ataxia) | chr11 | 126269024 | 126278131 | 126219024 | 126328131 | 109107 |  |
| *FRRS1L* | CeGaT diagnostic panels (neurodegeneration) | chr9 | 109130293 | 109167249 | 109080293 | 109217249 | 136956 |  |
| *FTL* | MDS Taskforce list (chorea) | chr19 | 48965309 | 48966879 | 48915309 | 49016879 | 101570 | Twist Alliance Dark Genes |
| *FUCA1* | CeGaT diagnostic panels (neurodegeneration) | chr1 | 23845077 | 23868290 | 23795077 | 23918290 | 123213 | Twist Alliance Dark Genes |
| *FUS* | CeGaT diagnostic panels (neurodegeneration) | chr16 | 31180138 | 31196963 | 31130138 | 31246963 | 116825 |  |
| *FXN* | MDS Taskforce list (ataxia) | chr9 | 69035751 | 69079076 | 68985751 | 69129076 | 143325 | Twist Alliance Dark Genes; Challenging medically relevant gene (CMRG) |
| *FYN* | GWAS loci (PD) | chr6 | 111660332 | 111873452 | 111610332 | 111923452 | 313120 |  |
| *GAK* | GWAS loci (PD) | chr4 | 849276 | 932373 | 799276 | 982373 | 183097 | Twist Alliance Dark Genes; Challenging medically relevant gene (CMRG) |
| *GALC* | MDS Taskforce list (ataxia) | chr14 | 87837820 | 87993665 | 87787820 | 88043665 | 255845 |  |
| *GAN* | MDS Taskforce list (ataxia) | chr16 | 81314944 | 81390809 | 81264944 | 81440809 | 175865 |  |
| *GBA1* | GWAS loci (PD) | chr1 | 155234452 | 155244699 | 155184452 | 155294699 | 110247 | Twist Alliance Dark Genes |
| *GBA2* | MDS Taskforce list (ataxia) | chr9 | 35736866 | 35749228 | 35686866 | 35799228 | 112362 |  |
| *GBE1* | CeGaT diagnostic panels (neurodegeneration) | chr3 | 81489703 | 81761645 | 81439703 | 81811645 | 371942 |  |
| *GBF1* | GWAS loci (PD) | chr10 | 102245371 | 102382899 | 102195371 | 102432899 | 237528 |  |
| *GCDH* | MDS Taskforce list (chorea) | chr19 | 12891128 | 12915126 | 12841128 | 12965126 | 123998 |  |
| *GCH1* | MDS Taskforce list (dystonia) | chr14 | 54842008 | 54902826 | 54792008 | 54952826 | 160818 |  |
| *GEMIN4* | CeGaT diagnostic panels (neurodegeneration) | chr17 | 744421 | 753999 | 694421 | 803999 | 109578 |  |
| *GFAP* | MDS Taskforce list (ataxia) | chr17 | 44903159 | 44916937 | 44853159 | 44966937 | 113778 |  |
| *GFM1* | CeGaT diagnostic panels (neurodegeneration) | chr3 | 158644527 | 158695581 | 158594527 | 158745581 | 151054 |  |
| *GIGYF2* | CeGaT diagnostic panels (neurodegeneration) | chr2 | 232697299 | 232860605 | 232647299 | 232910605 | 263306 |  |
| *GIPC1* | Other repeat expansion loci | chr19 | 14477760 | 14496149 | 14427760 | 14546149 | 118389 |  |
| *GJC2* | MDS Taskforce list (ataxia) | chr1 | 228149930 | 228159826 | 228099930 | 228209826 | 109896 |  |
| *GLA* | CeGaT diagnostic panels (neurodegeneration) | chrX | 101393273 | 101408012 | 101343273 | 101458012 | 114739 |  |
| *GLB1* | MDS Taskforce list (ataxia) | chr3 | 32996609 | 33097202 | 32946609 | 33147202 | 200593 |  |
| *GLDC* | CeGaT diagnostic panels (neurodegeneration) | chr9 | 6532467 | 6645729 | 6482467 | 6695729 | 213262 |  |
| *GLRX5* | CeGaT diagnostic panels (neurodegeneration) | chr14 | 95533503 | 95544724 | 95483503 | 95594724 | 111221 |  |
| *GMPPB* | CeGaT diagnostic panels (neurodegeneration) | chr3 | 49716844 | 49723973 | 49666844 | 49773973 | 107129 |  |
| *GNAL* | MDS Taskforce list (dystonia) | chr18 | 11689264 | 11885685 | 11639264 | 11935685 | 296421 |  |
| *GNAO1* | MDS Taskforce list (chorea) | chr16 | 56189660 | 56357444 | 56139660 | 56407444 | 267784 |  |
| *GNB1* | CeGaT diagnostic panels (neurodegeneration) | chr1 | 1785285 | 1892292 | 1735285 | 1942292 | 207007 |  |
| *GOSR2* | MDS Taskforce list (ataxia) | chr17 | 46923075 | 46975524 | 46873075 | 47025524 | 152449 |  |
| *GPNMB* | GWAS loci (PD) | chr7 | 23235967 | 23275108 | 23185967 | 23325108 | 139141 |  |
| *GRID2* | MDS Taskforce list (ataxia) | chr4 | 92303966 | 93810157 | 92253966 | 93860157 | 1606191 |  |
| *GRM1* | CeGaT diagnostic panels (neurodegeneration) | chr6 | 146027646 | 146437601 | 145977646 | 146487601 | 509955 |  |
| *GRN* | MDS Taskforce list (ataxia) | chr17 | 44345246 | 44353106 | 44295246 | 44403106 | 107860 |  |
| *HEPACAM* | MDS Taskforce list (ataxia) | chr11 | 124919205 | 124936412 | 124869205 | 124986412 | 117207 |  |
| *HEXA* | MDS Taskforce list (ataxia) | chr15 | 72340924 | 72376420 | 72290924 | 72426420 | 135496 |  |
| *HEXB* | MDS Taskforce list (ataxia) | chr5 | 74640023 | 74722647 | 74590023 | 74772647 | 182624 |  |
| *HIKESHI* | CeGaT diagnostic panels (neurodegeneration) | chr11 | 86302211 | 86345943 | 86252211 | 86395943 | 143732 |  |
| *HIP1R* | GWAS loci (PD) | chr12 | 122834453 | 122862961 | 122784453 | 122912961 | 128508 |  |
| *HLA-DRB5* | GWAS loci (PD) | chr6 | 32517353 | 32530287 | 32467353 | 32580287 | 112934 | MHC region |
| *HNRNPA1* | CeGaT diagnostic panels (neurodegeneration) | chr12 | 54280193 | 54287088 | 54230193 | 54337088 | 106895 |  |
| *HPCA* | MDS Taskforce list (dystonia) | chr1 | 32885994 | 32898441 | 32835994 | 32948441 | 112447 |  |
| *HPRT1* | MDS Taskforce list (chorea) | chrX | 134460165 | 134520531 | 134410165 | 134570531 | 160366 |  |
| *HSD17B4* | MDS Taskforce list (ataxia) | chr5 | 119452465 | 119637199 | 119402465 | 119687199 | 284734 |  |
| *HSPD1* | CeGaT diagnostic panels (neurodegeneration) | chr2 | 197486584 | 197516737 | 197436584 | 197566737 | 130153 |  |
| *HTRA1* | CeGaT diagnostic panels (neurodegeneration) | chr10 | 122458551 | 122514907 | 122408551 | 122564907 | 156356 |  |
| *HTRA2* | CeGaT diagnostic panels (neurodegeneration) | chr2 | 74529596 | 74533350 | 74479596 | 74583350 | 103754 |  |
| *HTT* | MDS Taskforce list (chorea) | chr4 | 3041363 | 3243957 | 2991363 | 3293957 | 302594 |  |
| *HYCC1* | CeGaT diagnostic panels (neurodegeneration) | chr7 | 22889371 | 23014130 | 22839371 | 23064130 | 224759 |  |
| *IBA57* | CeGaT diagnostic panels (neurodegeneration) | chr1 | 228165804 | 228182257 | 228115804 | 228232257 | 116453 |  |
| *IDS* | CeGaT diagnostic panels (neurodegeneration) | chrX | 149476988 | 149521096 | 149426988 | 149571096 | 144108 |  |
| *IFIH1* | CeGaT diagnostic panels (neurodegeneration) | chr2 | 162267074 | 162318684 | 162217074 | 162368684 | 151610 |  |
| *IGSF9B* | GWAS loci (PD) | chr11 | 133896438 | 133956968 | 133846438 | 134006968 | 160530 |  |
| *INPP5F* | GWAS loci (PD) | chr10 | 119726042 | 119829147 | 119676042 | 119879147 | 203105 |  |
| *IP6K2* | GWAS loci (PD) | chr3 | 48688003 | 48740353 | 48638003 | 48790353 | 152350 |  |
| *IRF2BPL* | MDS Taskforce list (dystonia) | chr14 | 77024543 | 77028708 | 76974543 | 77078708 | 104165 |  |
| *ISCA1* | CeGaT diagnostic panels (neurodegeneration) | chr9 | 86264546 | 86283102 | 86214546 | 86333102 | 118556 |  |
| *ISCA2* | CeGaT diagnostic panels (neurodegeneration) | chr14 | 74493756 | 74497106 | 74443756 | 74547106 | 103350 |  |
| *ITGA8* | GWAS loci (PD) | chr10 | 15513954 | 15719922 | 15463954 | 15769922 | 305968 |  |
| *ITM2B* | CeGaT diagnostic panels (neurodegeneration) | chr13 | 48232612 | 48270357 | 48182612 | 48320357 | 137745 |  |
| *ITPKB* | GWAS loci (PD) | chr1 | 226631690 | 226739323 | 226581690 | 226789323 | 207633 |  |
| *ITPR1* | MDS Taskforce list (ataxia) | chr3 | 4493345 | 4847506 | 4443345 | 4897506 | 454161 |  |
| *JAM2* | MDS Taskforce list (PD) | chr21 | 25639258 | 25717562 | 25589258 | 25767562 | 178304 |  |
| *JPH3* | MDS Taskforce list (chorea) | chr16 | 87601835 | 87698156 | 87551835 | 87748156 | 196321 |  |
| *KARS1* | CeGaT diagnostic panels (neurodegeneration) | chr16 | 75627474 | 75648643 | 75577474 | 75698643 | 121169 |  |
| *KCNA1* | CeGaT diagnostic panels (neurodegeneration) | chr12 | 4909905 | 4918256 | 4859905 | 4968256 | 108351 |  |
| *KCNC3* | MDS Taskforce list (ataxia) | chr19 | 50311937 | 50333515 | 50261937 | 50383515 | 121578 |  |
| *KCND3* | MDS Taskforce list (ataxia) | chr1 | 111770662 | 111989668 | 111720662 | 112039668 | 319006 |  |
| *KCNIP3* | GWAS loci (PD) | chr2 | 95297327 | 95386077 | 95247327 | 95436077 | 188750 |  |
| *KCNJ10* | MDS Taskforce list (ataxia) | chr1 | 159998651 | 160070483 | 159948651 | 160120483 | 171832 |  |
| *KCNMA1* | CeGaT diagnostic panels (neurodegeneration) | chr10 | 76869601 | 77638369 | 76819601 | 77688369 | 868768 |  |
| *KCNN2* | CeGaT diagnostic panels (neurodegeneration) | chr5 | 114055926 | 114496500 | 114005926 | 114546500 | 540574 |  |
| *KCNS3* | GWAS loci (PD) | chr2 | 17877847 | 18361616 | 17827847 | 18411616 | 583769 |  |
| *KCNT1* | CeGaT diagnostic panels (neurodegeneration) | chr9 | 135702185 | 135795508 | 135652185 | 135845508 | 193323 | Twist Alliance Dark Genes |
| *KCTD17* | MDS Taskforce list (dystonia) | chr22 | 37051739 | 37063390 | 37001739 | 37113390 | 111651 |  |
| *KCTD7* | MDS Taskforce list (ataxia) | chr7 | 66628881 | 66649067 | 66578881 | 66699067 | 120186 |  |
| *KIDINS220* | CeGaT diagnostic panels (neurodegeneration) | chr2 | 8721081 | 8837630 | 8671081 | 8887630 | 216549 |  |
| *KIF1A* | MDS Taskforce list (ataxia) | chr2 | 240713761 | 240824293 | 240663761 | 240874293 | 210532 |  |
| *KIF1C* | MDS Taskforce list (ataxia) | chr17 | 4997950 | 5028401 | 4947950 | 5078401 | 130451 |  |
| *KIF5A* | CeGaT diagnostic panels (neurodegeneration) | chr12 | 57546026 | 57586633 | 57496026 | 57636633 | 140607 |  |
| *KMT2B* | MDS Taskforce list (dystonia) | chr19 | 35717973 | 35738880 | 35667973 | 35788880 | 120907 |  |
| *KPNA1* | GWAS loci (PD) | chr3 | 122421902 | 122514945 | 122371902 | 122564945 | 193043 |  |
| *KRTCAP2* | GWAS loci (PD) | chr1 | 155169408 | 155173475 | 155119408 | 155223475 | 104067 |  |
| *L1CAM* | CeGaT diagnostic panels (neurodegeneration) | chrX | 153861514 | 153886173 | 153811514 | 153936173 | 124659 |  |
| *L2HGDH* | MDS Taskforce list (ataxia) | chr14 | 50237563 | 50312229 | 50187563 | 50362229 | 174666 |  |
| *LAMA2* | CeGaT diagnostic panels (neurodegeneration) | chr6 | 128883138 | 129516566 | 128833138 | 129566566 | 733428 |  |
| *LARGE1* | CeGaT diagnostic panels (neurodegeneration) | chr22 | 33162226 | 33922841 | 33112226 | 33972841 | 860615 |  |
| *LCORL* | GWAS loci (PD) | chr4 | 17841187 | 18021876 | 17791187 | 18071876 | 280689 |  |
| *LGALS3* | CeGaT diagnostic panels (neurodegeneration) | chr14 | 55124110 | 55145423 | 55074110 | 55195423 | 121313 |  |
| *LINC02210-CRHR1* | CeGaT diagnostic panels (neurodegeneration) | chr17 | 45620344 | 45835826 | 45570344 | 45885826 | 315482 |  |
| *LIPT2* | CeGaT diagnostic panels (neurodegeneration) | chr11 | 74490519 | 74493724 | 74440519 | 74543724 | 103205 |  |
| *LMBRD1* | CeGaT diagnostic panels (neurodegeneration) | chr6 | 69672757 | 69867236 | 69622757 | 69917236 | 294479 |  |
| *LMNB1* | MDS Taskforce list (ataxia) | chr5 | 126776623 | 126837020 | 126726623 | 126887020 | 160397 |  |
| *LRP10* | CeGaT diagnostic panels (neurodegeneration) | chr14 | 22871740 | 22881713 | 22821740 | 22931713 | 109973 |  |
| *LRP12* | Other repeat expansion loci | chr8 | 104489231 | 104589258 | 104439231 | 104639258 | 200027 |  |
| *LRPPRC* | MDS Taskforce list (ataxia) | chr2 | 43886224 | 43996226 | 43836224 | 44046226 | 210002 |  |
| *LRRK2* | MDS Taskforce list (PD) | chr12 | 40196744 | 40369285 | 40146744 | 40419285 | 272541 |  |
| *LRRK2-DT* | CeGaT diagnostic panels (neurodegeneration) | chr12 | 40196766 | 40224915 | 40146766 | 40274915 | 128149 |  |
| *LYRM7* | CeGaT diagnostic panels (neurodegeneration) | chr5 | 131170918 | 131205428 | 131120918 | 131255428 | 134510 |  |
| *MAG* | MDS Taskforce list (ataxia) | chr19 | 35291647 | 35316351 | 35241647 | 35366351 | 124704 |  |
| *MAP4K4* | GWAS loci (PD) | chr2 | 101696850 | 101894690 | 101646850 | 101944690 | 297840 |  |
| *MAPT* | MDS Taskforce list (PD) | chr17 | 45894358 | 46028334 | 45844358 | 46078334 | 233976 |  |
| *MARCHF6* | Other repeat expansion loci | chr5 | 10353695 | 10440388 | 10303695 | 10490388 | 186693 |  |
| *MARS2* | CeGaT diagnostic panels (neurodegeneration) | chr2 | 197705369 | 197708395 | 197655369 | 197758395 | 103026 |  |
| *MATR3* | CeGaT diagnostic panels (neurodegeneration) | chr5 | 139273752 | 139331671 | 139223752 | 139381671 | 157919 |  |
| *MBNL2* | GWAS loci (PD) | chr13 | 97221434 | 97394120 | 97171434 | 97444120 | 272686 |  |
| *MCCC1* | GWAS loci (PD) | chr3 | 183015218 | 183116075 | 182965218 | 183166075 | 200857 |  |
| *MCOLN1* | CeGaT diagnostic panels (neurodegeneration) | chr19 | 7522624 | 7534009 | 7472624 | 7584009 | 111385 |  |
| *MECR* | MDS Taskforce list (dystonia) | chr1 | 29192657 | 29230942 | 29142657 | 29280942 | 138285 |  |
| *MED12L* | GWAS loci (PD) | chr3 | 151085286 | 151437072 | 151035286 | 151487072 | 451786 |  |
| *MEX3C* | GWAS loci (PD) | chr18 | 51174550 | 51218333 | 51124550 | 51268333 | 143783 |  |
| *MFSD8* | MDS Taskforce list (ataxia) | chr4 | 127917799 | 127966034 | 127867799 | 128016034 | 148235 |  |
| *MIPOL1* | GWAS loci (PD) | chr14 | 37197894 | 37579125 | 37147894 | 37629125 | 481231 |  |
| *MLC1* | MDS Taskforce list (ataxia) | chr22 | 50059391 | 50085426 | 50009391 | 50135426 | 126035 | Twist Alliance Dark Genes; Challenging medically relevant gene (CMRG) |
| *MMUT* | MDS Taskforce list (chorea) | chr6 | 49430360 | 49463253 | 49380360 | 49513253 | 132893 |  |
| *MPI* | CeGaT diagnostic panels (neurodegeneration) | chr15 | 74890005 | 74902219 | 74840005 | 74952219 | 112214 |  |
| *MPV17* | MDS Taskforce list (ataxia) | chr2 | 27309492 | 27325680 | 27259492 | 27375680 | 116188 |  |
| *MRE11* | MDS Taskforce list (ataxia) | chr11 | 94415570 | 94493885 | 94365570 | 94543885 | 178315 |  |
| *MRPL43* | CeGaT diagnostic panels (neurodegeneration) | chr10 | 100969458 | 100987515 | 100919458 | 101037515 | 118057 |  |
| *MRPS34* | CeGaT diagnostic panels (neurodegeneration) | chr16 | 1771890 | 1773150 | 1721890 | 1823150 | 101260 |  |
| *mtDNA* | CeGaT diagnostic panels (neurodegeneration) | chrM | 1 | 16569 | *NA* | *NA* | 16569 |  |
| *MTFMT* | MDS Taskforce list (ataxia) | chr15 | 65001512 | 65029639 | 64951512 | 65079639 | 128127 |  |
| *MTHFS* | CeGaT diagnostic panels (neurodegeneration) | chr15 | 79833585 | 79897379 | 79783585 | 79947379 | 163794 |  |
| *MTRFR* | CeGaT diagnostic panels (neurodegeneration) | chr12 | 123233385 | 123258079 | 123183385 | 123308079 | 124694 |  |
| *MTTP* | MDS Taskforce list (ataxia) | chr4 | 99564081 | 99623997 | 99514081 | 99673997 | 159916 |  |
| *MUC1* | CeGaT diagnostic panels (neurodegeneration) | chr1 | 155185824 | 155192916 | 155135824 | 155242916 | 107092 | Twist Alliance Dark Genes; Challenging medically relevant gene (CMRG) |
| *MYORG* | CeGaT diagnostic panels (neurodegeneration) | chr9 | 34366666 | 34376898 | 34316666 | 34426898 | 110232 |  |
| *NACC1* | CeGaT diagnostic panels (neurodegeneration) | chr19 | 13116862 | 13141147 | 13066862 | 13191147 | 124285 |  |
| *NARS2* | CeGaT diagnostic panels (neurodegeneration) | chr11 | 78435620 | 78575194 | 78385620 | 78625194 | 239574 |  |
| *NAXE* | CeGaT diagnostic panels (neurodegeneration) | chr1 | 156591756 | 156609507 | 156541756 | 156659507 | 117751 |  |
| *NDUFS1* | CeGaT diagnostic panels (neurodegeneration) | chr2 | 206114817 | 206159509 | 206064817 | 206209509 | 144692 |  |
| *NDUFV1* | CeGaT diagnostic panels (neurodegeneration) | chr11 | 67605653 | 67612554 | 67555653 | 67662554 | 106901 |  |
| *NEU1* | MDS Taskforce list (ataxia) | chr6 | 31857659 | 31862905 | 31807659 | 31912905 | 105246 | MHC region |
| *NFU1* | CeGaT diagnostic panels (neurodegeneration) | chr2 | 69396113 | 69437628 | 69346113 | 69487628 | 141515 |  |
| *NIPA1* | Other repeat expansion loci | chr15 | 22773063 | 22829789 | 22723063 | 22879789 | 156726 |  |
| *NKX2-1* | MDS Taskforce list (chorea) | chr14 | 36516392 | 36521149 | 36466392 | 36571149 | 104757 |  |
| *NKX6-2* | CeGaT diagnostic panels (neurodegeneration) | chr10 | 132783179 | 132786147 | 132733179 | 132836147 | 102968 |  |
| *NOD2* | GWAS loci (PD) | chr16 | 50693588 | 50733077 | 50643588 | 50783077 | 139489 |  |
| *NOP56* | MDS Taskforce list (ataxia) | chr20 | 2652593 | 2658393 | 2602593 | 2708393 | 105800 |  |
| *NOTCH2NLC* | Other repeat expansion loci | chr1 | 149390621 | 149471833 | 149340621 | 149521833 | 181212 |  |
| *NOTCH3* | CeGaT diagnostic panels (neurodegeneration) | chr19 | 15159038 | 15200995 | 15109038 | 15250995 | 141957 |  |
| *NPC1* | MDS Taskforce list (ataxia) | chr18 | 23506184 | 23586506 | 23456184 | 23636506 | 180322 |  |
| *NPC2* | MDS Taskforce list (ataxia) | chr14 | 74476192 | 74494177 | 74426192 | 74544177 | 117985 |  |
| *NR4A2* | CeGaT diagnostic panels (neurodegeneration) | chr2 | 156324437 | 156342348 | 156274437 | 156392348 | 117911 |  |
| *NT5C2* | CeGaT diagnostic panels (neurodegeneration) | chr10 | 103087185 | 103277605 | 103037185 | 103327605 | 290420 |  |
| *NUBPL* | MDS Taskforce list (ataxia) | chr14 | 31489956 | 31861224 | 31439956 | 31911224 | 471268 |  |
| *NUCKS1* | GWAS loci (PD) | chr1 | 205712822 | 205750182 | 205662822 | 205800182 | 137360 |  |
| *NUTM2B* | CeGaT diagnostic panels (neurodegeneration) | chr10 | 79703227 | 79714681 | 79653227 | 79764681 | 111454 | Twist Alliance Dark Genes; Challenging medically relevant gene (CMRG) |
| *OCLN* | CeGaT diagnostic panels (neurodegeneration) | chr5 | 69492292 | 69558104 | 69442292 | 69608104 | 165812 | Twist Alliance Dark Genes; Challenging medically relevant gene (CMRG) |
| *OPA3* | CeGaT diagnostic panels (neurodegeneration) | chr19 | 45527767 | 45602212 | 45477767 | 45652212 | 174445 |  |
| *OPTN* | CeGaT diagnostic panels (neurodegeneration) | chr10 | 13099449 | 13138308 | 13049449 | 13188308 | 138859 |  |
| *PABPN1* | Other repeat expansion loci | chr14 | 23321457 | 23326163 | 23271457 | 23376163 | 104706 |  |
| *PAM* | GWAS loci (PD) | chr5 | 102753981 | 103029730 | 102703981 | 103079730 | 375749 |  |
| *PANK2* | MDS Taskforce list (ataxia) | chr20 | 3888839 | 3929887 | 3838839 | 3979887 | 141048 |  |
| *PARK7* | MDS Taskforce list (PD) | chr1 | 7954291 | 7985505 | 7904291 | 8035505 | 131214 |  |
| *PC* | MDS Taskforce list (ataxia) | chr11 | 66848417 | 66958386 | 66798417 | 67008386 | 209969 |  |
| *PCCA* | MDS Taskforce list (chorea) | chr13 | 100089015 | 100530437 | 100039015 | 100580437 | 541422 |  |
| *PCCB* | MDS Taskforce list (chorea) | chr3 | 136250340 | 136337896 | 136200340 | 136387896 | 187556 | Twist Alliance Dark Genes; Challenging medically relevant gene (CMRG) |
| *PDE10A* | MDS Taskforce list (chorea) | chr6 | 165327287 | 165988117 | 165277287 | 166038117 | 760830 |  |
| *PDE8B* | MDS Taskforce list (PD) | chr5 | 77086732 | 77428256 | 77036732 | 77478256 | 441524 |  |
| *PDGFB* | CeGaT diagnostic panels (neurodegeneration) | chr22 | 39223359 | 39244982 | 39173359 | 39294982 | 121623 |  |
| *PDGFRB* | MDS Taskforce list (PD) | chr5 | 150113839 | 150155872 | 150063839 | 150205872 | 142033 |  |
| *PDHA1* | CeGaT diagnostic panels (neurodegeneration) | chrX | 19343893 | 19361718 | 19293893 | 19411718 | 117825 |  |
| *PDYN* | MDS Taskforce list (ataxia) | chr20 | 1978757 | 1994285 | 1928757 | 2044285 | 115528 |  |
| *PET100* | CeGaT diagnostic panels (neurodegeneration) | chr19 | 7629788 | 7631956 | 7579788 | 7681956 | 102168 |  |
| *PEX1* | MDS Taskforce list (ataxia) | chr7 | 92487020 | 92528520 | 92437020 | 92578520 | 141500 |  |
| *PEX10* | MDS Taskforce list (ataxia) | chr1 | 2403964 | 2413797 | 2353964 | 2463797 | 109833 |  |
| *PEX11B* | CeGaT diagnostic panels (neurodegeneration) | chr1 | 145911350 | 145918717 | 145861350 | 145968717 | 107367 |  |
| *PEX12* | CeGaT diagnostic panels (neurodegeneration) | chr17 | 35574795 | 35578863 | 35524795 | 35628863 | 104068 |  |
| *PEX13* | CeGaT diagnostic panels (neurodegeneration) | chr2 | 61017225 | 61051990 | 60967225 | 61101990 | 134765 |  |
| *PEX14* | CeGaT diagnostic panels (neurodegeneration) | chr1 | 10472288 | 10630758 | 10422288 | 10680758 | 258470 |  |
| *PEX16* | CeGaT diagnostic panels (neurodegeneration) | chr11 | 45909663 | 45918812 | 45859663 | 45968812 | 109149 |  |
| *PEX19* | CeGaT diagnostic panels (neurodegeneration) | chr1 | 160276807 | 160286348 | 160226807 | 160336348 | 109541 |  |
| *PEX2* | MDS Taskforce list (ataxia) | chr8 | 76980258 | 77001044 | 76930258 | 77051044 | 120786 |  |
| *PEX26* | CeGaT diagnostic panels (neurodegeneration) | chr22 | 18077923 | 18105396 | 18027923 | 18155396 | 127473 |  |
| *PEX3* | CeGaT diagnostic panels (neurodegeneration) | chr6 | 143450805 | 143490616 | 143400805 | 143540616 | 139811 |  |
| *PEX5* | CeGaT diagnostic panels (neurodegeneration) | chr12 | 7188685 | 7218574 | 7138685 | 7268574 | 129889 | Twist Alliance Dark Genes; Challenging medically relevant gene (CMRG) |
| *PEX6* | MDS Taskforce list (ataxia) | chr6 | 42963865 | 42979181 | 42913865 | 43029181 | 115316 |  |
| *PEX7* | MDS Taskforce list (ataxia) | chr6 | 136822564 | 136913934 | 136772564 | 136963934 | 191370 |  |
| *PFN1* | CeGaT diagnostic panels (neurodegeneration) | chr17 | 4945652 | 4949061 | 4895652 | 4999061 | 103409 | Twist Alliance Dark Genes |
| *PHGDH* | CeGaT diagnostic panels (neurodegeneration) | chr1 | 119648411 | 119744218 | 119598411 | 119794218 | 195807 |  |
| *PHYH* | MDS Taskforce list (ataxia) | chr10 | 13277796 | 13302412 | 13227796 | 13352412 | 124616 |  |
| *PINK1* | MDS Taskforce list (PD) | chr1 | 20633458 | 20651511 | 20583458 | 20701511 | 118053 |  |
| *PLA2G6* | MDS Taskforce list (ataxia) | chr22 | 38111495 | 38214778 | 38061495 | 38264778 | 203283 |  |
| *PLAA* | CeGaT diagnostic panels (neurodegeneration) | chr9 | 26903372 | 26947242 | 26853372 | 26997242 | 143870 |  |
| *PLEKHG2* | CeGaT diagnostic panels (neurodegeneration) | chr19 | 39412669 | 39428415 | 39362669 | 39478415 | 115746 |  |
| *PLP1* | MDS Taskforce list (ataxia) | chrX | 103773718 | 103792619 | 103723718 | 103842619 | 118901 |  |
| *PMPCA* | MDS Taskforce list (ataxia) | chr9 | 136410630 | 136427992 | 136360630 | 136477992 | 117362 |  |
| *PMPCB* | CeGaT diagnostic panels (neurodegeneration) | chr7 | 103297407 | 103331818 | 103247407 | 103381818 | 134411 |  |
| *PMVK* | GWAS loci (PD) | chr1 | 154924740 | 154936719 | 154874740 | 154986719 | 111979 |  |
| *PNKD* | CeGaT diagnostic panels (neurodegeneration) | chr2 | 218269651 | 218346793 | 218219651 | 218396793 | 177142 |  |
| *PNKP* | MDS Taskforce list (ataxia) | chr19 | 49859882 | 49878351 | 49809882 | 49928351 | 118469 | Twist Alliance Dark Genes; Challenging medically relevant gene (CMRG) |
| *PNPLA6* | MDS Taskforce list (ataxia) | chr19 | 7534004 | 7561764 | 7484004 | 7611764 | 127760 |  |
| *POLG* | CeGaT diagnostic panels (neurodegeneration) | chr15 | 89305198 | 89334861 | 89255198 | 89384861 | 129663 |  |
| *POLG2* | CeGaT diagnostic panels (neurodegeneration) | chr17 | 64477785 | 64497054 | 64427785 | 64547054 | 119269 | Twist Alliance Dark Genes; Challenging medically relevant gene (CMRG) |
| *POLR1C* | MDS Taskforce list (ataxia) | chr6 | 43509702 | 43562419 | 43459702 | 43612419 | 152717 |  |
| *POLR3A* | MDS Taskforce list (ataxia) | chr10 | 77953148 | 78029522 | 77903148 | 78079522 | 176374 |  |
| *POLR3B* | MDS Taskforce list (ataxia) | chr12 | 106357748 | 106510198 | 106307748 | 106560198 | 252450 |  |
| *POLR3K* | CeGaT diagnostic panels (neurodegeneration) | chr16 | 46407 | 53608 | -3593 | 103608 | 107201 |  |
| *POMGNT1* | CeGaT diagnostic panels (neurodegeneration) | chr1 | 46188683 | 46220305 | 46138683 | 46270305 | 131622 |  |
| *POMT1* | CeGaT diagnostic panels (neurodegeneration) | chr9 | 131502789 | 131523806 | 131452789 | 131573806 | 121017 |  |
| *POMT2* | CeGaT diagnostic panels (neurodegeneration) | chr14 | 77274956 | 77320883 | 77224956 | 77370883 | 145927 |  |
| *PPP2R2B* | MDS Taskforce list (ataxia) | chr5 | 146580742 | 147084784 | 146530742 | 147134784 | 604042 |  |
| *PPT1* | CeGaT diagnostic panels (neurodegeneration) | chr1 | 40072710 | 40097260 | 40022710 | 40147260 | 124550 |  |
| *PRICKLE1* | CeGaT diagnostic panels (neurodegeneration) | chr12 | 42456757 | 42590355 | 42406757 | 42640355 | 233598 |  |
| *PRKCG* | MDS Taskforce list (ataxia) | chr19 | 53879190 | 53907652 | 53829190 | 53957652 | 128462 | Twist Alliance Dark Genes; Challenging medically relevant gene (CMRG) |
| *PRKN* | CeGaT diagnostic panels (neurodegeneration) | chr6 | 161347417 | 162727775 | 161297417 | 162777775 | 1480358 |  |
| *PRKRA* | MDS Taskforce list (dystonia) | chr2 | 178431292 | 178451512 | 178381292 | 178501512 | 120220 |  |
| *PRNP* | Other repeat expansion loci | chr20 | 4686350 | 4701590 | 4636350 | 4751590 | 115240 |  |
| *PRRT2* | CeGaT diagnostic panels (neurodegeneration) | chr16 | 29811382 | 29815892 | 29761382 | 29865892 | 104510 |  |
| *PSAP* | CeGaT diagnostic panels (neurodegeneration) | chr10 | 71816298 | 71851251 | 71766298 | 71901251 | 134953 |  |
| *PSAT1* | CeGaT diagnostic panels (neurodegeneration) | chr9 | 78297125 | 78330093 | 78247125 | 78380093 | 132968 |  |
| *PSEN1* | MDS Taskforce list (ataxia) | chr14 | 73136418 | 73223691 | 73086418 | 73273691 | 187273 |  |
| *PSEN2* | CeGaT diagnostic panels (neurodegeneration) | chr1 | 226870184 | 226927726 | 226820184 | 226977726 | 157542 |  |
| *PTPN1* | CeGaT diagnostic panels (neurodegeneration) | chr20 | 50510321 | 50585241 | 50460321 | 50635241 | 174920 |  |
| *PTRHD1* | CeGaT diagnostic panels (neurodegeneration) | chr2 | 24789728 | 24793391 | 24739728 | 24843391 | 103663 |  |
| *PTS* | MDS Taskforce list (dystonia) | chr11 | 112226367 | 112269955 | 112176367 | 112319955 | 143588 |  |
| *PUM1* | MDS Taskforce list (ataxia) | chr1 | 30931506 | 31065991 | 30881506 | 31115991 | 234485 |  |
| *PYCR2* | CeGaT diagnostic panels (neurodegeneration) | chr1 | 225919877 | 225924340 | 225869877 | 225974340 | 104463 |  |
| *QARS1* | CeGaT diagnostic panels (neurodegeneration) | chr3 | 49095932 | 49105130 | 49045932 | 49155130 | 109198 |  |
| *QDPR* | MDS Taskforce list (dystonia) | chr4 | 17460261 | 17512206 | 17410261 | 17562206 | 151945 |  |
| *RAB11B* | CeGaT diagnostic panels (neurodegeneration) | chr19 | 8389981 | 8404434 | 8339981 | 8454434 | 114453 |  |
| *RAB12* | CeGaT diagnostic panels (neurodegeneration) | chr18 | 8609437 | 8639383 | 8559437 | 8689383 | 129946 |  |
| *RAB29* | GWAS loci (PD) | chr1 | 205767986 | 205775482 | 205717986 | 205825482 | 107496 |  |
| *RAB32* | MDS Taskforce list (PD) | chr6 | 146543833 | 146554953 | 146493833 | 146604953 | 111120 |  |
| *RAB39B* | MDS Taskforce list (PD) | chrX | 155258235 | 155264491 | 155208235 | 155314491 | 106256 |  |
| *RAPGEF2* | Other repeat expansion loci | chr4 | 159103013 | 159360174 | 159053013 | 159410174 | 357161 |  |
| *RARS1* | CeGaT diagnostic panels (neurodegeneration) | chr5 | 168486451 | 168519301 | 168436451 | 168569301 | 132850 |  |
| *RARS2* | CeGaT diagnostic panels (neurodegeneration) | chr6 | 87513459 | 87590028 | 87463459 | 87640028 | 176569 |  |
| *REEP1* | MDS Taskforce list (ataxia) | chr2 | 86213993 | 86338083 | 86163993 | 86388083 | 224090 |  |
| *REEP2* | CeGaT diagnostic panels (neurodegeneration) | chr5 | 138439057 | 138446969 | 138389057 | 138496969 | 107912 |  |
| *RETREG3* | GWAS loci (PD) | chr17 | 42579513 | 42610623 | 42529513 | 42660623 | 131110 |  |
| *RFC1* | MDS Taskforce list (ataxia) | chr4 | 39287456 | 39366375 | 39237456 | 39416375 | 178919 |  |
| *RHOT1* | CeGaT diagnostic panels (neurodegeneration) | chr17 | 32142454 | 32253374 | 32092454 | 32303374 | 210920 |  |
| *RIC3* | CeGaT diagnostic panels (neurodegeneration) | chr11 | 8106056 | 8169055 | 8056056 | 8219055 | 162999 |  |
| *RIMS1* | GWAS loci (PD) | chr6 | 71886550 | 72403150 | 71836550 | 72453150 | 616600 |  |
| *RIT2* | GWAS loci (PD) | chr18 | 42743227 | 43115691 | 42693227 | 43165691 | 472464 |  |
| *RNASEH2A* | CeGaT diagnostic panels (neurodegeneration) | chr19 | 12806584 | 12813640 | 12756584 | 12863640 | 107056 |  |
| *RNASEH2B* | CeGaT diagnostic panels (neurodegeneration) | chr13 | 50909747 | 51024120 | 50859747 | 51074120 | 214373 |  |
| *RNASEH2C* | CeGaT diagnostic panels (neurodegeneration) | chr11 | 65714005 | 65720818 | 65664005 | 65770818 | 106813 |  |
| *RNASET2* | CeGaT diagnostic panels (neurodegeneration) | chr6 | 166922113 | 166957191 | 166872113 | 167007191 | 135078 |  |
| *RNF141* | GWAS loci (PD) | chr11 | 10511673 | 10541230 | 10461673 | 10591230 | 129557 |  |
| *RNF170* | CeGaT diagnostic panels (neurodegeneration) | chr8 | 42849637 | 42897290 | 42799637 | 42947290 | 147653 |  |
| *RNF216* | MDS Taskforce list (ataxia) | chr7 | 5620047 | 5781696 | 5570047 | 5831696 | 261649 |  |
| *RPS12* | GWAS loci (PD) | chr6 | 132814569 | 132817564 | 132764569 | 132867564 | 102995 |  |
| *RPS6KL1* | GWAS loci (PD) | chr14 | 74903951 | 74923302 | 74853951 | 74973302 | 119351 |  |
| *RTN2* | CeGaT diagnostic panels (neurodegeneration) | chr19 | 45485294 | 45497055 | 45435294 | 45547055 | 111761 |  |
| *SACS* | MDS Taskforce list (ataxia) | chr13 | 23288689 | 23433763 | 23238689 | 23483763 | 245074 |  |
| *SAMD12* | Other repeat expansion loci | chr8 | 118189455 | 118622112 | 118139455 | 118672112 | 532657 |  |
| *SAMHD1* | CeGaT diagnostic panels (neurodegeneration) | chr20 | 36890229 | 36951893 | 36840229 | 37001893 | 161664 |  |
| *SATB1* | GWAS loci (PD) | chr3 | 18345377 | 18445621 | 18295377 | 18495621 | 200244 |  |
| *SCAF11* | GWAS loci (PD) | chr12 | 45919131 | 45992120 | 45869131 | 46042120 | 172989 |  |
| *SCARB2* | MDS Taskforce list (ataxia) | chr4 | 76158737 | 76234536 | 76108737 | 76284536 | 175799 |  |
| *SCN1A* | CeGaT diagnostic panels (neurodegeneration) | chr2 | 165984641 | 166182806 | 165934641 | 166232806 | 298165 |  |
| *SCN2A* | CeGaT diagnostic panels (neurodegeneration) | chr2 | 165194993 | 165392310 | 165144993 | 165442310 | 297317 |  |
| *SCN8A* | CeGaT diagnostic panels (neurodegeneration) | chr12 | 51590266 | 51812864 | 51540266 | 51862864 | 322598 |  |
| *SCP2* | CeGaT diagnostic panels (neurodegeneration) | chr1 | 52927276 | 53051698 | 52877276 | 53101698 | 224422 |  |
| *SCYL1* | CeGaT diagnostic panels (neurodegeneration) | chr11 | 65525077 | 65538704 | 65475077 | 65588704 | 113627 |  |
| *SDHA* | MDS Taskforce list (ataxia) | chr5 | 218303 | 257082 | 168303 | 307082 | 138779 | Twist Alliance Dark Genes; Challenging medically relevant gene (CMRG) |
| *SDHAF1* | CeGaT diagnostic panels (neurodegeneration) | chr19 | 35995188 | 35996312 | 35945188 | 36046312 | 101124 |  |
| *SETD1A* | GWAS loci (PD) | chr16 | 30957294 | 30984664 | 30907294 | 31034664 | 127370 |  |
| *SETX* | MDS Taskforce list (ataxia) | chr9 | 132261356 | 132354986 | 132211356 | 132404986 | 193630 |  |
| *SGCE* | MDS Taskforce list (dystonia) | chr7 | 94524204 | 94656572 | 94474204 | 94706572 | 232368 |  |
| *SH3GL2* | GWAS loci (PD) | chr9 | 17579066 | 17797124 | 17529066 | 17847124 | 318058 |  |
| *SIGMAR1* | CeGaT diagnostic panels (neurodegeneration) | chr9 | 34634722 | 34637844 | 34584722 | 34687844 | 103122 | Twist Alliance Dark Genes |
| *SIL1* | MDS Taskforce list (ataxia) | chr5 | 138946724 | 139293557 | 138896724 | 139343557 | 446833 |  |
| *SIPA1L2* | GWAS loci (PD) | chr1 | 232397965 | 232630571 | 232347965 | 232680571 | 332606 |  |
| *SLC16A2* | MDS Taskforce list (ataxia) | chrX | 74421493 | 74533917 | 74371493 | 74583917 | 212424 |  |
| *SLC17A5* | MDS Taskforce list (ataxia) | chr6 | 73593379 | 73653992 | 73543379 | 73703992 | 160613 | Twist Alliance Dark Genes; Challenging medically relevant gene (CMRG) |
| *SLC19A3* | MDS Taskforce list (ataxia) | chr2 | 227683763 | 227718028 | 227633763 | 227768028 | 134265 |  |
| *SLC1A3* | CeGaT diagnostic panels (neurodegeneration) | chr5 | 36596588 | 36688334 | 36546588 | 36738334 | 191746 |  |
| *SLC1A4* | CeGaT diagnostic panels (neurodegeneration) | chr2 | 64988477 | 65023865 | 64938477 | 65073865 | 135388 |  |
| *SLC20A2* | MDS Taskforce list (PD) | chr8 | 42416475 | 42541926 | 42366475 | 42591926 | 225451 |  |
| *SLC25A12* | CeGaT diagnostic panels (neurodegeneration) | chr2 | 171783405 | 171999859 | 171733405 | 172049859 | 316454 |  |
| *SLC2A1* | MDS Taskforce list (ataxia) | chr1 | 42925353 | 42958893 | 42875353 | 43008893 | 133540 |  |
| *SLC30A10* | MDS Taskforce list (dystonia) | chr1 | 219685427 | 219959018 | 219635427 | 220009018 | 373591 |  |
| *SLC33A1* | MDS Taskforce list (ataxia) | chr3 | 155821024 | 155854456 | 155771024 | 155904456 | 133432 |  |
| *SLC39A14* | MDS Taskforce list (dystonia) | chr8 | 22367278 | 22434129 | 22317278 | 22484129 | 166851 |  |
| *SLC6A3* | MDS Taskforce list (dystonia) | chr5 | 1392794 | 1445440 | 1342794 | 1495440 | 152646 | Twist Alliance Dark Genes; Challenging medically relevant gene (CMRG) |
| *SNCA* | MDS Taskforce list (PD) | chr4 | 89700345 | 89838315 | 89650345 | 89888315 | 237970 |  |
| *SNCAIP* | GWAS loci (PD) | chr5 | 122311354 | 122464219 | 122261354 | 122514219 | 252865 |  |
| *SNORD118* | MDS Taskforce list (ataxia) | chr17 | 8173454 | 8173587 | 8123454 | 8223587 | 100133 |  |
| *SNX14* | MDS Taskforce list (ataxia) | chr6 | 85504776 | 85594156 | 85454776 | 85644156 | 189380 |  |
| *SOD1* | CeGaT diagnostic panels (neurodegeneration) | chr21 | 31659666 | 31668931 | 31609666 | 31718931 | 109265 |  |
| *SOX10* | CeGaT diagnostic panels (neurodegeneration) | chr22 | 37970686 | 37987422 | 37920686 | 38037422 | 116736 |  |
| *SPART* | MDS Taskforce list (ataxia) | chr13 | 36301638 | 36370180 | 36251638 | 36420180 | 168542 |  |
| *SPAST* | CeGaT diagnostic panels (neurodegeneration) | chr2 | 32063556 | 32157637 | 32013556 | 32207637 | 194081 |  |
| *SPG11* | MDS Taskforce list (ataxia) | chr15 | 44554818 | 44663688 | 44504818 | 44713688 | 208870 |  |
| *SPG21* | MDS Taskforce list (ataxia) | chr15 | 64963022 | 64990310 | 64913022 | 65040310 | 127288 |  |
| *SPG7* | MDS Taskforce list (ataxia) | chr16 | 89490719 | 89557766 | 89440719 | 89607766 | 167047 | Twist Alliance Dark Genes |
| *SPPL2B* | GWAS loci (PD) | chr19 | 2328615 | 2355095 | 2278615 | 2405095 | 126480 |  |
| *SPR* | MDS Taskforce list (ataxia) | chr2 | 72887382 | 72892158 | 72837382 | 72942158 | 104776 |  |
| *SPTAN1* | CeGaT diagnostic panels (neurodegeneration) | chr9 | 128552558 | 128633662 | 128502558 | 128683662 | 181104 |  |
| *SPTBN2* | MDS Taskforce list (ataxia) | chr11 | 66682496 | 66744670 | 66632496 | 66794670 | 162174 |  |
| *SPTSSB* | GWAS loci (PD) | chr3 | 161344798 | 161372880 | 161294798 | 161422880 | 128082 |  |
| *SQSTM1* | MDS Taskforce list (ataxia) | chr5 | 179806398 | 179838078 | 179756398 | 179888078 | 131680 |  |
| *STARD7* | Other repeat expansion loci | chr2 | 96184859 | 96208827 | 96134859 | 96258827 | 123968 |  |
| *STK39* | GWAS loci (PD) | chr2 | 167954020 | 168247595 | 167904020 | 168297595 | 393575 |  |
| *STN1* | CeGaT diagnostic panels (neurodegeneration) | chr10 | 103856806 | 103918332 | 103806806 | 103968332 | 161526 |  |
| *STUB1* | MDS Taskforce list (ataxia) | chr16 | 680224 | 682870 | 630224 | 732870 | 102646 |  |
| *SUCLA2* | MDS Taskforce list (dystonia) | chr13 | 47745736 | 48037968 | 47695736 | 48087968 | 392232 |  |
| *SUMF1* | MDS Taskforce list (ataxia) | chr3 | 3700814 | 4467273 | 3650814 | 4517273 | 866459 |  |
| *SURF1* | CeGaT diagnostic panels (neurodegeneration) | chr9 | 133351758 | 133356676 | 133301758 | 133406676 | 104918 |  |
| *SYNE1* | MDS Taskforce list (ataxia) | chr6 | 152121687 | 152637801 | 152071687 | 152687801 | 616114 |  |
| *SYNJ1* | MDS Taskforce list (PD) | chr21 | 32628759 | 32728040 | 32578759 | 32778040 | 199281 |  |
| *SYT17* | GWAS loci (PD) | chr16 | 19167971 | 19268332 | 19117971 | 19318332 | 200361 |  |
| *TACO1* | CeGaT diagnostic panels (neurodegeneration) | chr17 | 63600882 | 63608365 | 63550882 | 63658365 | 107483 |  |
| *TAF1* | CeGaT diagnostic panels (neurodegeneration) | chrX | 71366136 | 71532374 | 71316136 | 71582374 | 266238 |  |
| *TARDBP* | CeGaT diagnostic panels (neurodegeneration) | chr1 | 11012344 | 11030528 | 10962344 | 11080528 | 118184 | Twist Alliance Dark Genes |
| *TARS2* | CeGaT diagnostic panels (neurodegeneration) | chr1 | 150487414 | 150507602 | 150437414 | 150557602 | 120188 |  |
| *TBCD* | CeGaT diagnostic panels (neurodegeneration) | chr17 | 82752042 | 82945914 | 82702042 | 82995914 | 293872 |  |
| *TBCK* | CeGaT diagnostic panels (neurodegeneration) | chr4 | 106041599 | 106316683 | 105991599 | 106366683 | 375084 |  |
| *TBK1* | CeGaT diagnostic panels (neurodegeneration) | chr12 | 64452090 | 64502114 | 64402090 | 64552114 | 150024 |  |
| *TBP* | MDS Taskforce list (ataxia) | chr6 | 170554302 | 170572870 | 170504302 | 170622870 | 118568 |  |
| *TDP1* | MDS Taskforce list (ataxia) | chr14 | 89954939 | 90044764 | 89904939 | 90094764 | 189825 |  |
| *TDP2* | CeGaT diagnostic panels (neurodegeneration) | chr6 | 24649979 | 24666930 | 24599979 | 24716930 | 116951 |  |
| *TECPR2* | CeGaT diagnostic panels (neurodegeneration) | chr14 | 102362941 | 102502477 | 102312941 | 102552477 | 239536 |  |
| *TFAM* | CeGaT diagnostic panels (neurodegeneration) | chr10 | 58385345 | 58399220 | 58335345 | 58449220 | 113875 |  |
| *TFG* | CeGaT diagnostic panels (neurodegeneration) | chr3 | 100709295 | 100748964 | 100659295 | 100798964 | 139669 |  |
| *TGFB1* | CeGaT diagnostic panels (neurodegeneration) | chr19 | 41301587 | 41353922 | 41251587 | 41403922 | 152335 |  |
| *TGM6* | MDS Taskforce list (ataxia) | chr20 | 2380901 | 2432753 | 2330901 | 2482753 | 151852 |  |
| *TH* | MDS Taskforce list (dystonia) | chr11 | 2163929 | 2171815 | 2113929 | 2221815 | 107886 |  |
| *THAP1* | MDS Taskforce list (dystonia) | chr8 | 42836674 | 42843325 | 42786674 | 42893325 | 106651 |  |
| *TIMM8A* | MDS Taskforce list (dystonia) | chrX | 101345661 | 101348742 | 101295661 | 101398742 | 103081 |  |
| *TMEM106B* | CeGaT diagnostic panels (neurodegeneration) | chr7 | 12211270 | 12243367 | 12161270 | 12293367 | 132097 |  |
| *TMEM163* | GWAS loci (PD) | chr2 | 134455759 | 134719000 | 134405759 | 134769000 | 363241 |  |
| *TMEM175* | GWAS loci (PD) | chr4 | 932387 | 958656 | 882387 | 1008656 | 126269 | Twist Alliance Dark Genes |
| *TMEM230* | CeGaT diagnostic panels (neurodegeneration) | chr20 | 5068232 | 5113087 | 5018232 | 5163087 | 144855 |  |
| *TMEM240* | MDS Taskforce list (ataxia) | chr1 | 1534778 | 1540624 | 1484778 | 1590624 | 105846 |  |
| *TNRC6A* | Other repeat expansion loci | chr16 | 24610209 | 24827632 | 24560209 | 24877632 | 317423 |  |
| *TOR1A* | MDS Taskforce list (dystonia) | chr9 | 129812942 | 129824244 | 129762942 | 129874244 | 111302 |  |
| *TPP1* | MDS Taskforce list (ataxia) | chr11 | 6612768 | 6619448 | 6562768 | 6669448 | 106680 |  |
| *TRAPPC12* | CeGaT diagnostic panels (neurodegeneration) | chr2 | 3379675 | 3485094 | 3329675 | 3535094 | 205419 |  |
| *TRAPPC6B* | CeGaT diagnostic panels (neurodegeneration) | chr14 | 39147811 | 39170532 | 39097811 | 39220532 | 122721 |  |
| *TRAPPC9* | CeGaT diagnostic panels (neurodegeneration) | chr8 | 139727725 | 140458579 | 139677725 | 140508579 | 830854 |  |
| *TREM2* | CeGaT diagnostic panels (neurodegeneration) | chr6 | 41158506 | 41163186 | 41108506 | 41213186 | 104680 |  |
| *TREX1* | CeGaT diagnostic panels (neurodegeneration) | chr3 | 48465811 | 48467645 | 48415811 | 48517645 | 101834 |  |
| *TRIM40* | GWAS loci (PD) | chr6 | 30136124 | 30148735 | 30086124 | 30198735 | 112611 | MHC region |
| *TTBK2* | MDS Taskforce list (ataxia) | chr15 | 42738730 | 42920809 | 42688730 | 42970809 | 282079 |  |
| *TTC19* | MDS Taskforce list (ataxia) | chr17 | 15999784 | 16045015 | 15949784 | 16095015 | 145231 |  |
| *TTPA* | MDS Taskforce list (ataxia) | chr8 | 63048553 | 63086053 | 62998553 | 63136053 | 137500 |  |
| *TUBA4A* | CeGaT diagnostic panels (neurodegeneration) | chr2 | 219249710 | 219277902 | 219199710 | 219327902 | 128192 |  |
| *TUBB4A* | MDS Taskforce list (dystonia) | chr19 | 6494319 | 6502848 | 6444319 | 6552848 | 108529 |  |
| *TUFM* | CeGaT diagnostic panels (neurodegeneration) | chr16 | 28842411 | 28846348 | 28792411 | 28896348 | 103937 | Twist Alliance Dark Genes |
| *TWNK* | CeGaT diagnostic panels (neurodegeneration) | chr10 | 100987367 | 100994403 | 100937367 | 101044403 | 107036 |  |
| *TYMP* | CeGaT diagnostic panels (neurodegeneration) | chr22 | 50525752 | 50530032 | 50475752 | 50580032 | 104280 |  |
| *TYROBP* | CeGaT diagnostic panels (neurodegeneration) | chr19 | 35904401 | 35908295 | 35854401 | 35958295 | 103894 |  |
| *UBAP1* | CeGaT diagnostic panels (neurodegeneration) | chr9 | 34179005 | 34252523 | 34129005 | 34302523 | 173518 |  |
| *UBAP2* | GWAS loci (PD) | chr9 | 33921693 | 34049388 | 33871693 | 34099388 | 227695 |  |
| *UBQLN2* | CeGaT diagnostic panels (neurodegeneration) | chrX | 56563627 | 56567868 | 56513627 | 56617868 | 104241 |  |
| *UBTF* | GWAS loci (PD) | chr17 | 44205033 | 44221626 | 44155033 | 44271626 | 116593 |  |
| *UCHL1* | MDS Taskforce list (ataxia) | chr4 | 41256413 | 41268455 | 41206413 | 41318455 | 112042 |  |
| *UFC1* | CeGaT diagnostic panels (neurodegeneration) | chr1 | 161152776 | 161158856 | 161102776 | 161208856 | 106080 |  |
| *UFM1* | CeGaT diagnostic panels (neurodegeneration) | chr13 | 38349849 | 38363619 | 38299849 | 38413619 | 113770 |  |
| *UQCRC1* | CeGaT diagnostic panels (neurodegeneration) | chr3 | 48599002 | 48610976 | 48549002 | 48660976 | 111974 |  |
| *VAC14* | MDS Taskforce list (dystonia) | chr16 | 70687439 | 70801160 | 70637439 | 70851160 | 213721 | Twist Alliance Dark Genes |
| *VAMP1* | MDS Taskforce list (ataxia) | chr12 | 6462237 | 6470677 | 6412237 | 6520677 | 108440 |  |
| *VAMP4* | GWAS loci (PD) | chr1 | 171700160 | 171742074 | 171650160 | 171792074 | 141914 |  |
| *VAPB* | CeGaT diagnostic panels (neurodegeneration) | chr20 | 58389229 | 58451101 | 58339229 | 58501101 | 161872 |  |
| *VARS1* | CeGaT diagnostic panels (neurodegeneration) | chr6 | 31777518 | 31795752 | 31727518 | 31845752 | 118234 | MHC region |
| *VARS2* | MDS Taskforce list (ataxia) | chr6 | 30914205 | 30926459 | 30864205 | 30976459 | 112254 | MHC region |
| *VCP* | CeGaT diagnostic panels (neurodegeneration) | chr9 | 35053928 | 35072668 | 35003928 | 35122668 | 118740 |  |
| *VLDLR* | MDS Taskforce list (ataxia) | chr9 | 2621182 | 2660056 | 2571182 | 2710056 | 138874 |  |
| *VPS11* | CeGaT diagnostic panels (neurodegeneration) | chr11 | 119067818 | 119081972 | 119017818 | 119131972 | 114154 |  |
| *VPS13A* | MDS Taskforce list (chorea) | chr9 | 77177445 | 77421537 | 77127445 | 77471537 | 344092 |  |
| *VPS13C* | MDS Taskforce list (PD) | chr15 | 61852389 | 62060473 | 61802389 | 62110473 | 308084 |  |
| *VPS13D* | MDS Taskforce list (ataxia) | chr1 | 12230030 | 12512047 | 12180030 | 12562047 | 382017 |  |
| *VPS16* | MDS Taskforce list (dystonia) | chr20 | 2840703 | 2866732 | 2790703 | 2916732 | 126029 |  |
| *VPS35* | MDS Taskforce list (PD) | chr16 | 46656132 | 46689518 | 46606132 | 46739518 | 133386 | Twist Alliance Dark Genes |
| *VWA1* | Other repeat expansion loci | chr1 | 1434861 | 1442882 | 1384861 | 1492882 | 108021 |  |
| *WARS2* | CeGaT diagnostic panels (neurodegeneration) | chr1 | 119031216 | 119140654 | 118981216 | 119190654 | 209438 |  |
| *WASHC5* | CeGaT diagnostic panels (neurodegeneration) | chr8 | 125024260 | 125091819 | 124974260 | 125141819 | 167559 |  |
| *WDR45* | MDS Taskforce list (dystonia) | chrX | 49074433 | 49101170 | 49024433 | 49151170 | 126737 |  |
| *WDR45B* | CeGaT diagnostic panels (neurodegeneration) | chr17 | 82614562 | 82648553 | 82564562 | 82698553 | 133991 |  |
| *WDR81* | MDS Taskforce list (ataxia) | chr17 | 1716523 | 1738599 | 1666523 | 1788599 | 122076 |  |
| *WFS1* | MDS Taskforce list (ataxia) | chr4 | 6269849 | 6303265 | 6219849 | 6353265 | 133416 |  |
| *WNT3* | GWAS loci (PD) | chr17 | 46762506 | 46833154 | 46712506 | 46883154 | 170648 |  |
| *WWOX* | CeGaT diagnostic panels (neurodegeneration) | chr16 | 78099400 | 79212667 | 78049400 | 79262667 | 1213267 |  |
| *XK* | MDS Taskforce list (chorea) | chrX | 37685791 | 37732130 | 37635791 | 37782130 | 146339 |  |
| *XPR1* | MDS Taskforce list (PD) | chr1 | 180632022 | 180890279 | 180582022 | 180940279 | 358257 |  |
| *XRCC1* | MDS Taskforce list (ataxia) | chr19 | 43543311 | 43580473 | 43493311 | 43630473 | 137162 |  |
| *ZFHX3* | Other repeat expansion loci | chr16 | 72782885 | 73891871 | 72732885 | 73941871 | 1208986 |  |
| *ZFYVE26* | MDS Taskforce list (ataxia) | chr14 | 67727374 | 67816590 | 67677374 | 67866590 | 189216 |  |
| *ZFYVE27* | CeGaT diagnostic panels (neurodegeneration) | chr10 | 97737121 | 97760907 | 97687121 | 97810907 | 123786 |  |
| *ZNHIT3* | CeGaT diagnostic panels (neurodegeneration) | chr17 | 36486629 | 36499310 | 36436629 | 36549310 | 112681 |  |

**Twist Alliance Dark Genes**: Medically-relevant genes known for segmental duplications/high sequence similarity with pseudogenes that are hard to sequence using short reads. Mahmoud M, Harting J, Corbitt H, et al. Closing the gap: Solving complex medically relevant genes at scale. Preprint. medRxiv. 2024;2024.03.14.24304179. Published 2024 Mar 18.

**Challenging medically relevant gene (CMRG)**: Medically-relevant genes (CMRGs) are situated in complex or highly repetitive regions of the human genome. Ji, Y., Zhao, J., Gong, J. et al. Unveiling novel genetic variants in 370 challenging medically relevant genes using the long-read sequencing data of 41 samples from 19 global populations. Mol Genet Genomics 299, 65 (2024). https://doi.org/10.1007/s00438-024-02158-x.

**Supplementary Table 2. Read statistics from the nanopore long-read adaptive sampling runs**.

| Sample ID | Barcode | Run | Wash steps | Total bases (Mb) | Mean quality (Q-score) | Total reads | Mean read length (bp) | Total mapped On-target reads | Mean On-target read N50 (bp) | Mean Off-target read N50 (bp) | Mean On-target coverage (X) | Mean Off-target coverage (X) | On/Off fold change |
| --- | --- | --- | --- | --- | --- | --- | --- | --- | --- | --- | --- | --- | --- |
| L-15936 | BC96 | AS-04 | 1 | 7555 | 19.7 | 8,711,030 | 3005 | 356,272 | 16,023 | 620 | 15.7 | 1.6 | 9.8 |
| SFC817 | BC94 | AS-04 | 1 | 10,819 | 19.1 | 9,277,700 | 6237 | 418,805 | 35,941 | 649 | 42.9 | 1.9 | 22.6 |
| iPS-L-3244 | BC90 | AS-02 | 1 | 9431 | 18.0 | 9,008,980 | 4963 | 416,703 | 30,383 | 647 | 30.6 | 1.9 | 16.1 |
| iPS-L-3034 | BC1 | AS-01 | 0 | 1654 | 21.3 | 986,800 | 11,479 | 119,448 | 36,040 | 792 | 19.5 | 1.0 | 19.5 |
| L-13754 | BC89 | AS-02 | 1 | 9334 | 16.9 | 9,192,520 | 4493 | 387,097 | 20,407 | 627 | 28.0 | 1.9 | 14.7 |
| L-26618 | BC88 | AS-02 | 1 | 9326 | 17.8 | 8,945,940 | 4948 | 377,404 | 22,670 | 646 | 29.5 | 1.8 | 16.4 |
| SFC831 | BC3 | AS-01 | 0 | 1108 | 20.8 | 693,580 | 9368 | 86,644 | 36,853 | 777 | 12.6 | 0.7 | 18.0 |
| L-10180 | BC93 | AS-03 | 1 | 4388 | 15.9 | 4,724,320 | 3113 | 178,735 | 14,144 | 636 | 11.1 | 0.9 | 12.3 |
| L-20456 | BC62 | AS-07 | 2 | 10,861 | 17.9 | 15,315,140 | 1290 | 559,414 | 5,661 | 543 | 14.2 | 2.4 | 5.9 |
| L-20468 | BC92 | AS-03 | 1 | 14,491 | 16.8 | 20,077,750 | 1247 | 661,108 | 5,908 | 613 | 17.0 | 3.3 | 5.2 |
| L-20677 | BC75 | AS-05 | 2 | 13,729 | 20.9 | 15,637,280 | 3900 | 696,387 | 16,003 | 662 | 29.3 | 3.1 | 9.5 |
| D298 | BC81 | AS-05 | 2 | 8501 | 21.8 | 8,815,100 | 7326 | 361,219 | 15,769 | 678 | 22.5 | 1.9 | 11.8 |
| L-14372 | BC64 | AS-07 | 2 | 13,435 | 19.0 | 19,225,720 | 1363 | 694,450 | 5,761 | 559 | 17.8 | 3.0 | 5.9 |
| L-20799 | BC82 | AS-05 | 2 | 19,401 | 20.5 | 23,450,220 | 3111 | 1,053,562 | 10,880 | 656 | 40.0 | 4.4 | 9.1 |
| L-15764 | BC95 | AS-04 | 1 | 8887 | 18.6 | 8,144,320 | 5925 | 353,202 | 27,655 | 642 | 30.6 | 1.8 | 17.0 |
| L-1689 | BC86 | AS-06 | 2 | 9472 | 23.4 | 12,064,720 | 2393 | 535,476 | 16,777 | 573 | 20.9 | 1.8 | 11.6 |
| L-4474 | BC76 | AS-06 | 2 | 4618 | 22.3 | 3,138,310 | 10,611 | 119,630 | 34,205 | 666 | 20.6 | 0.7 | 29.4 |
| L-8319 | BC85 | AS-06 | 2 | 6220 | 22.4 | 4,145,840 | 11,942 | 166,252 | 35,825 | 666 | 28.9 | 0.9 | 32.1 |
| Mean | | | | 9068 | 19.6 | 10,086,404 | 5373 | 418,989 | 21,495 | 647 | 24.0 | 1.9 | 14.8 |
| Median | | | | 9330 | 19.4 | 8,977,460 | 4721 | 382,251 | 18,592 | 647 | 21.7 | 1.9 | 13.5 |
| SD | | | | 4566 | 2.2 | 6,501,365 | 3485 | 251,251 | 11,287 | 62 | 9.0 | 1.0 | 7.6 |
| IQR | | | | 4641 | 3.4 | 10,590,820 | 4321 | 380,679 | 20,061 | 46 | 12.5 | 1.4 | 8.5 |

Descriptive statistics were calculated based on all 18 samples of the panel. SD: standard deviation, IQR: interquartile range, kb: kilobase, Q-score: read quality (Phred), X: depth of coverage.

**Supplementary Table 3. List of Primer sequences used for the validation of additional pathogenic variants**.

| **Gene** | **Forward Primer (5'->3')** | **Reverse Primer (5'->3')** |
| --- | --- | --- |
| *ALS2* | AATCTAAGTCATCTGTGGCC | GCCAAGCCAGAAATTTATCC |
| *CNBP* | GAGATCATACCACTGCACTC | AGTATTACTGCCAGTGTGTG |
| *STXBP2* | CTGCAAGGTTCTCTCATCAG | CTCCAGGTTACGGATGAGG |
| *AP4S1* | TTCAAGGTTCCAGTTACAGC | CTTGTTCATTGGATCGAGAG |
| *RARS2* | ACGTACCTTCTCTGCTAGTC | ACACATAACACCTTCTTCTGG |

**Supplementary Table 4. Overview of additional false-positive variants**.

| Sample ID | Chromosome | GRCh38 Position | Ref | Alt | Variant type | Gene | Genotype | Validation |
| --- | --- | --- | --- | --- | --- | --- | --- | --- |
| L-10180 | Chr15 | 44,613,499-44,613,500 | C | CT | SNV | *SPG11*, c.3075dup | Het. | negative |
| L-10180 | Chr21 | 31,701,023 | GT | G | SNV | *SCAF4*, c.750del | Het. | negative |
| L-10180 | Chr18 | 23,539,436 | C | T | SNV | *NPC1*, c.2830G>A | Het. | negative |
| L-10180 | Chr4 | 99,583,407 | C | T | SNV | *MTTP*, c.283C>T | Het. | negative |
| L-10180 | Chr12 | 57,631,320 | GC | G | SNV | *B4GALNT1*, c.263del | Het. | negative |
| L-20468 | Chr19 | 12,891,966 | G | A | SNV | *GCDH*, c.263G>A | Het. | negative |
| L-26617 | Chr1 | 150,553,106 | T | TC | SNV | *ADAMTSL4*, c.292dup | Het. | negative |
| SFC817 | Chr13 | 110,677,070-110,677,319 | - | - | SV | *CARS2*, 250 bp deletion (exon7) | Het. | negative |
| L-20456 | Chr13 | 70,139,384-70,139,428 | - | - | STR | *ATXN8OS*, (CTG)_150_ | Het. | negative |

Filtering of the EPI2ME output yielded 14 not previously reported candidate variants, nine variants of which could not be validated using either Sanger sequencing or PCR and are listed here. SNV: single nucleotide variant, SV: structural variant, STR: short tandem repeat, bp: base pairs, Het: heterozygous.

**Supplementary Table 5. *RFC1* repeat lengths**.

| Sample ID | Position | Mean *RFC1* coverage (X) | Repeat motif | Allele 1, size (bp) | Allele 1, RN | Allele 2, size (bp) | Allele 2, RN |
| --- | --- | --- | --- | --- | --- | --- | --- |
| SFC817 | chr4:39348425-39348479 | 45.3 | AAAAG | 620 | 124 | 50 | 10 |
| L-13754 | chr4:39348425-39348479 | 25.0 | AAAAG | 577 | 115 | 500 | 100 |
| L-26618 | chr4:39348425-39348479 | 28.9 | AAAAG | 504 | 101 | 50 | 10 |
| SFC831 | chr4:39348425-39348479 | 11.6 | AAAAG | 312 | 62 | 55 | 11 |
| L-10180 | chr4:39348425-39348479 | 8.9 | AAAAG | 550 | 110 | 49 | 10 |
| L-20456 | chr4:39348425-39348479 | 12.82 | AAAAG | 719 | 144 | 49 | 10 |
| L-20677 | chr4:39348425-39348479 | 30.5 | AAAAG | 313 | 63 | 55 | 11 |
| D298 | chr4:39348425-39348479 | 23.4 | AAAAG | 450 | 90 | 50 | 10 |
| L-20799 | chr4:39348425-39348479 | 42.1 | AAAAG | 590 | 118 | 532 | 106 |
| L-15764 | chr4:39348425-39348479 | 40.6 | AAAAG | 497 | 99 | 50 | 10 |
| L-1689 | chr4:39348425-39348479 | 19.7 | AAAAG | 285 | 57 | 50 | 10 |
| L-4474 | chr4:39348425-39348479 | 22.0 | AAAAG | 610 | 122 | 49 | 10 |
| L-8319 | chr4:39348425-39348479 | 29.7 | AAAAG | 675 | 135 | 549 | 109 |

The Panel comprised 18 individuals. Straglr, in the EPI2ME workflow, detected a benign RFC1 repeat expansion in 13 patients. L-13754, L-20799, and L-8319 carried the expansion on both alleles. bp: basepairs, X: depth of coverage, RN: Repeat number.

.

 **Supplementary Figure 1: NCRF results for L-1689**. NCRF mapped 45 reads to a reference of the *DAB1* repeat expansion with the motif “ATTTC”. Eight reads showed an expended repeat length with a median of 523, corresponding to a repeat number of ~105. NCRF: Noise Cancelling Repeat Finder.

**Supplementary Figure 2: NCRF results for L-15764**. NCRF mapped 672 reads to a reference of the *FGF14* repeat expansion with the motif “GAA”. 24 reads showed an expended repeat length (>600) with a median of 1045.5, corresponding to a repeat number of ~349. NCRF: Noise Cancelling Repeat Finder.

 **Supplementary Figure 3: NCRF results for L-20799**. NCRF mapped 47 reads to a reference of the *C9orf72* repeat expansion with the motif “GGGGCC”. 13 reads showed an expended repeat length (>1000). However, the repeat length varied to calculate a repeat number. NCRF: Noise Cancelling Repeat Finder.

**Supplementary Figure 4: IGV snapshot of the *PRKN* variants in iPS-L-3244.** The figure shows a snapshot from the Integrative Genomics Viewer (IGV) displaying haplotagged ONT reads mapped to Chromosome 6 of the GRCh38 reference genome. **A**: A heterozygous deletion of approximately 20 kb was identified in the region chr6:162710190-162729371, spanning exon 1 of *PRKN*. One read of haplotype 2 (blue) carries the deletion as highlighted by the mismatched bases. **B**: A SNV (*PRKN* c.823C>T) was detected at position chr6:161785820 on haplotype 1 (red). ONT: Oxford Nanopore Technologies.

**Supplementary Figure 5: IGV snapshot of the *PRKN* variants in L-13754**. The figure shows a snapshot from the Integrative Genomics Viewer (IGV) displaying haplotagged ONT reads mapped to Chromosome 6 of the GRCh38 reference genome. **A**: A heterozygous deletion of approximately 320 kb was identified in the region chr6:162236332-162551227, spanning exons 2-3 of *PRKN*. Mostly reads of haplotype 1 (red) remained. **B**: A small deletion (c.101_102del) was detected in position chr6:162443378 on haplotype 1 (red). The variant is located in the deleted stretch. ONT: Oxford Nanopore Technologies.

**Supplementary Figure 6: IGV snapshot of the *PRKN* deletions in iPS-L-3034**. The figure shows a snapshot from the Integrative Genomics Viewer (IGV) displaying ONT reads mapped to Chromosome 6 of the GRCh38 reference genome. Two compound heterozygous deletions of approximately 250 kb and 112 kb were identified, spanning exons 3-5 and exon 2 of *PRKN*, respectively. **A**: The left breakpoint of the 250 kb deletion at position chr6:162029818. Five reads of haplotype 1 (red) carry the deletion as highlighted by the mismatched bases. **B**: The right breakpoint of the 250 kb deletion at position chr6:162280411. Four reads of haplotype 1 (red) carry the deletion as highlighted by the mismatched bases. **C**: The 112 kb deletion in the region chr6:162336433-162448673. Eleven reads of haplotype 2 (blue) carry the deletion as highlighted by the mismatched bases. ONT: Oxford Nanopore Technologies.

**Supplementary Figure 7: IGV snapshot and Straglr result of the *C9orf72* repeat expansion in L-20799**. **A**. The figure shows a snapshot from the Integrative Genomics Viewer (IGV) displaying ONT reads mapped to Chromosome 9 of the GRCh38 reference genome. Six reads show an expansion of a length between 2,877 and 9,944 bp in *C9orf72*. **B**. The Straglr result of the EPI2ME human variation workflow also shows six reads with corresponding expanded repeat numbers. ONT: Oxford Nanopore Technologies.

**Supplementary Figure 8: IGV snapshot of the *TAF1* insertion in L-14372**. The figure shows a snapshot from the Integrative Genomics Viewer (IGV) displaying ONT reads mapped to Chromosome X of the GRCh38 reference genome. Three reads show an insertion of approximately 2.7 kb length in intron 32 of *TAF1*. ONT: Oxford Nanopore Technologies.

 **Supplementary Figure 9: Validation of SNVs and Indels**. Sanger Chromatograms illustrate the presence of three potentially pathogenic small variants in *STXBP2*, *AP4S1*, *RARS2*. **A**. A heterozygous G>C substitution at the position chr19:7645196 was confirmed in L-13754. **B**. A heterozygous C>T substitution at the position chr14:31066239 was confirmed in L-1689. **C**. A heterozygous ATT>T deletion at the position chr6:87564181 was confirmed in SFC817.

 **Supplementary Figure 10: Validation of SVs and repeat expansions**. Agarose Gels illustrate two potentially pathogenic variants in *ALS2* and *CNBP*. A. Lane 1 shows PCR products of an *ALS2* amplification in Sample iPS-L-3034. Two bands of the size ~2.1 kb and ~700 bp can be identified, highlighting the presence of a heterozygous 1.4 kb deletion in *ALS2*. The presence of the deletion was also validated in the native sample prior to iPSC reprogramming. Two markers were used, the New England Bioscience 1 kb DNA Ladder (left) and the New England Bioscience 100 bp DNA Ladder (right). B. Lane 1 shows PCR products of a *CNBP* amplification. Three bands, ~1.5 kb, ~900 bp, and ~700 bp in size, can be identified. The New England Bioscience 100 bp DNA Ladder was used (right).

**Supplementary Figure 11: NCRF results for L-15764**. NCRF mapped 922 reads to a reference of the *CNBP* repeat expansion with the motif “CCTG”. 12 reads showed an expended repeat length (>300) with a median of 563, corresponding to a repeat number of ~141. NCRF: Noise Cancelling Repeat Finder.

**Supplementary Figure 12: Use recommendations for our long-read adaptive sampling panel**. This exemplified diagnostic workflow illustrates potential use cases of the gene panel as a first- and second-line test. Prices are focused on raw sequencing and are highly dependent on geographical region, service provider, sequencing depth, and vary between research and clinical settings. The costs for each genetic test (per sample with ~20-30X Coverage) were estimated based on international studies (75-77), and on Chapter 11.4 of the EBM (German Uniform Evaluation Standard) catalog for the second quarter of 2026, provided by the National Association of Statutory Health Insurance Physicians (NASHIP) (78). The long-read sequencing costs are based on self-estimates for research purposes. PD: Parkinson's disease, HTT: Huntington's disease, NGS: Next generation sequencing, WES: Whole exome sequencing, WGS: Whole genome sequencing, BED: Browser Extensible Data, MLPA: Multiplex ligation-dependent probe amplification, SNV: Single nucleotide variant, SV: Structural variant, RED: Repeat expansion disorder. Created in BioRender. Fienemann, A. (2026) https://BioRender.com/nrxhmvb.

 **Supplementary Figure 13: Size distribution of 564 target regions included in the targeted long-read sequencing panel**. The size of the target regions, in kilobases (kb), is plotted against the counts. Each region is defined by the loci of interest ± 50 kb. Ten regions (*CAMTA1*, *ZFHX3*, *WWOX*, *PRKN*, *GRID2*, *DAB1*, and *DLG2*) exceeded 1 Mb in size.

 **Supplementary Figure 14: Chromosomal distribution of 564 target regions included in the targeted long-read sequencing panel**. The number of target regions located on each chromosome is plotted.

**Supplementary Figure 15: Cumulative read yield and pore occupancy over time**. The cumulative read yield and pore scan data are shown for all seven Flow cells (AS-01-AS-07) as reported by MinKNOW (version 24.06.15). Flow cell washing was either: not performed (**B**), performed once (**D**, **F**, **H**) or performed twice (**J**, **L**, **N**).
